# Loneliness in autistic adults: A systematic review

**DOI:** 10.1177/13623613221077721

**Published:** 2022-03-08

**Authors:** Kana Umagami, Anna Remington, Brynmor Lloyd-Evans, Jade Davies, Laura Crane

**Affiliations:** University College London, UK

**Keywords:** adults, autism, loneliness, relationships, systematic review

## Abstract

In this systematic review, we examined quantitative, qualitative and mixed methods studies on loneliness in autistic adults. A total of 1460 articles were identified, and 34 of these met inclusion criteria. Results demonstrated that (1) there is a paucity of qualitative data providing first-hand descriptions of loneliness from autistic adults; (2) few empirical studies have used reliable/valid measures of loneliness developed specifically for autistic adults, and in just one study was a measure of loneliness developed for, and validated in, autistic adults; (3) the collective dimension of loneliness (i.e. belonging in society) has been described by autistic adults, yet has not been investigated as frequently as the intimate (i.e. romantic relationships) or relational (i.e. friend/family relationships) dimensions of loneliness; (4) the factors associated with increased loneliness in autistic adults include autistic characteristics, anxiety, depression and suicidal ideation, negative experiences and learned helplessness, a lack of autism understanding and acceptance, sensory avoidance, camouflaging and unemployment; and (5) the factors associated with decreased loneliness in autistic adults include having relationships, participation in social skill interventions and/or experiencing fewer difficulties with social skills, positive views and acceptance of oneself, being female and time spent engaging in activities (e.g. online gaming). Directions for future research are considered.

**Lay abstract:**

Recently, researchers have been interested in how autistic people experience loneliness. Yet, most of this research has focused on loneliness in autistic children and young people. We present the results of a systematic review on loneliness in autistic adults. A systematic review is a rigorous way of searching for all existing research on a topic and summarizing the findings about specific questions. We searched for all research published on this topic until 9 April 2021. We found 34 articles that investigated loneliness in autistic adults. This research showed that (1) there is fairly little research that has involved directly asking autistic adults about their first-hand experiences of loneliness (e.g. what loneliness feels like for them); (2) few research studies have used loneliness questionnaires specifically developed for autistic adults (this was attempted in just one research study); (3) collective loneliness (i.e. loneliness associated with how much an autistic person feels they ‘fit in’ to society) seems important to autistic adults but has not been investigated as commonly as other aspects of loneliness (e.g. loneliness associated with romantic relationships or friendships); (4) things that might increase loneliness in autistic adults include anxiety and depression, and a lack of autism understanding and acceptance, for example; and (5) things that might reduce loneliness in autistic adults include having relationships and self-acceptance, for example. In our article, we discuss the kinds of future research on loneliness in autistic adults that might be useful.

Social isolation can include subjective and/or objective elements (Zavaleta & Samuel, 2014). Objective social isolation relates to the actual amount of social contact someone has, for example, less frequent social contact with others, having fewer people in one’s social network, and/or living alone (Holt-Lunstad et al., 2015). In contrast, subjective social isolation relates to the perceived adequacy of the quantity or quality of social relationships and incorporates concepts, such as perceived social support (Wang et al., 2017). Loneliness is a form of subjective social isolation and has been defined as a negative emotional state resulting from a gap between someone’s actual and desired social relationships (Peplau & Perlman, 1982). There is an ongoing debate as to whether loneliness is unidimensional or multidimensional. One of the most prominent multidimensional conceptualizations of loneliness was proposed by Cacioppo et al. (2015), who suggest that there are three dimensions of loneliness: intimate loneliness, which refers to the perceived absence of someone significant and emotionally close to the individual (e.g. a spouse); relational loneliness, which refers to the perceived absence of the people who are relatively close (e.g. friends, family) and collective loneliness, which refers to the perceived absence of belonging within larger groups in society (e.g. national identity). The consequences of loneliness are varied but often include physical and mental health problems (Cacioppo et al., 2006; Holt-Lunstad et al., 2010, 2015; O’Connell et al., 2004; Wang et al., 2018).

Loneliness has been relatively neglected in the autism research field, possibly due to early descriptions of autism emphasizing how autistic^1^ people prefer to be alone. Kanner (1943), for example, described one of his autistic patients as being happiest when he was left alone and observed autistic people’s ‘powerful desire for aloneness’ (p. 249). Furthermore, Asperger (1944, p. 38) noted that ‘human beings normally live in constant interaction with their environment and react to it continually. However, “autists” have severely disturbed and considerably limited interaction’.

Over time, these perceptions have changed. We know that many autistic people *are* interested in social connections with other people, despite sometimes experiencing difficulties with social interaction (Benford & Standen, 2009; Davidson, 2008). There has also been an increasing interest in research on loneliness in autistic people. Tending to focus on children and adolescents, research has shown that autistic children experience loneliness more intensely and more frequently than their non-autistic counterparts (Bauminger & Kasari, 2000; Bauminger et al., 2003). Autistic children also seem to experience loneliness qualitatively differently from their non-autistic peers. For example, studies have found that autistic children define loneliness solely based on being alone, while non-autistic children define loneliness in terms of both emotional and social-cognitive loneliness (Bauminger & Kasari, 2000). Other research has proposed a lack of friendship to be a key indicator of loneliness (Bauminger & Kasari, 2000; Locke et al., 2010), with many autistic children reported to have low levels of friendship quality and to be on the periphery of their school social networks (Calder et al., 2013; Locke et al., 2010). Although loneliness does not seem to be associated with an understanding of friendship among autistic or non-autistic children (Bottema-Beutel et al., 2019), low levels of friendship quality and/or being on the periphery of school social networks could lead to social withdrawal, isolation and loneliness in adolescence (Sumiya et al., 2018; White & Roberson-Nay, 2009; Whitehouse et al., 2009).

Little is known about the consequences of autistic people’s early experiences of loneliness. However, there are several reasons to suspect that loneliness will persist across the lifespan for autistic people. First, a lack of social relationships is often associated with loneliness, and difficulties with social interaction/communication and difficulties with social participation have been commonly reported in autistic adults (American Psychiatric Association, 2013; Myers et al., 2015). Second, once people grow up and are no longer in the mandatory social setting of school, the workplace could be a major source of social interaction preparation. Yet, research has consistently shown that autistic people have lower rates of employment than other disability groups (Office for National Statistics, 2021). Finally, support services for autistic individuals significantly decrease when they reach adulthood, with many autistic adults and their carers not being well informed about the social supports that are available to them (Anderson et al., 2018).

There is an emerging body of published research on loneliness in autistic adults, comprising quantitative, qualitative and mixed methods studies. These studies have examined a broad range of topics related to loneliness in autistic adults, including autistic people’s experiences of loneliness and the factors (positively and negatively) associated with loneliness. Given the breadth of the emerging research around this topic, it is essential to systematically identify the current evidence base, synthesize findings across studies and establish the extent of, and gaps in, current knowledge to guide priorities for future research. Conducting the first systematic review on loneliness in autistic adults, we aimed to identify quantitative and qualitative data on (1) autistic adults’ first-hand descriptions of loneliness; (2) how loneliness is measured and reported in studies on autistic adults; (3) the dimensions of loneliness (intimate, relational and collective) reported in research on autistic adults; (4) the factors reportedly associated with increased loneliness in autistic adults and (5) the factors reportedly associated with decreased loneliness in autistic adults (including interventions).

## Methods

This review, registered on the PROSPERO database (Registration No. CRD42019141853), adhered to the Preferred Reporting for Items for Systematic Reviews and Meta-Analyses (PRISMA) statement (Page et al., 2021).

### Search strategy

The search strategy was developed in consultation with a specialist librarian at IOE, UCL’s Faculty of Education and Society and through scoping searches of other autism-related systematic reviews (see Supplementary Appendix A). Articles were selected based on the relevance to the topic. In total, 12 sets of search words (autis* OR Asperger* OR Pervasive developmental disorder OR PDD OR ASD OR ASC) AND (lonel*OR social isolation) AND adult* plus one complete search term (autism AND loneliness AND adults) were used to adapt to the databases that did not respond to permutations of the words. Keywords ‘adult*’ and ‘adults’ were added to focus on the population of interest. On the advice of a specialist librarian, these words were searched as broadly as possible without applying limits to the search (i.e. searching within all fields) to bring up more relevant literature than when the searches were applied in limited ways (e.g. in keywords). The following bibliographic databases were searched: PsycINFO, Scopus, Education Resources Information Center (ERIC), Web of Science Core Collection, MEDLINE, British Education Index (BEI) and Applied Social Sciences Index and Abstracts (ASSIA). We conducted an initial search in early 2019, an updated search in early 2021 and a final search on 9 April 2021. The Cochrane library and PROSPERO were also searched to ensure no other systematic reviews on the topic existed. In addition to the bibliographic databases, dissertations/theses on the topic were searched through ProQuest Dissertations and Theses Global database. National and international experts in the field were contacted (in February 2019) to identify any work in progress/grey literature.

### Review criteria

Literature published in English from any country was included. Inclusion and exclusion criteria were focused on three domains: (1) diagnosis: studies were included when the results were separately reported for at least one autistic adult (formally diagnosed/self-identified^2^) and excluded when participants had high levels of autistic traits or were among the broader autism phenotype but were without an autism diagnosis; (2) age: studies were included when they specifically stated that they collected data from adult participants (even if the mean age or age range was not stated), or when the mean age of the adults was above 18 years and at least one adult participated in the study; and (3) study type: quantitative, qualitative and mixed method studies were included, including interventions; studies were excluded if they did not report data on loneliness. Dissertations/theses of any academic level were considered.

### Study selection process

**Figure 1.**
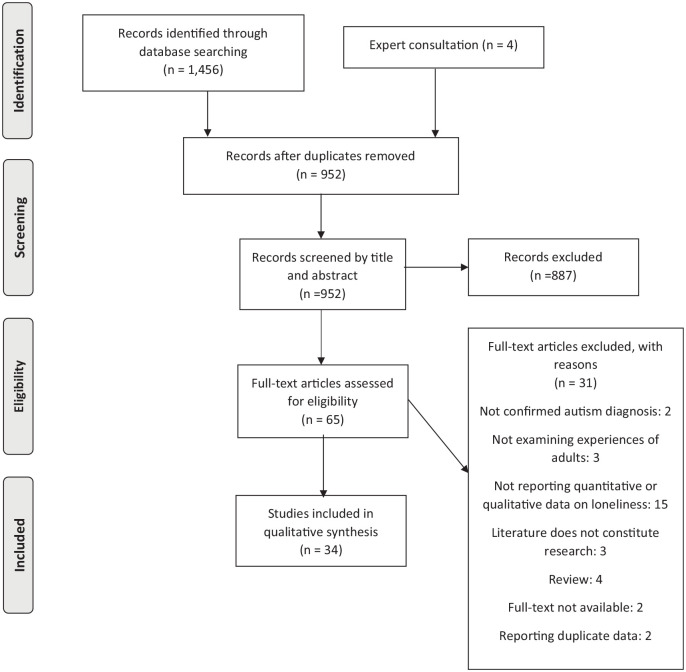
PRISMA diagram records identified through database searching.

After the initial database search, duplicates were removed using EndNote X9 and also by hand searching copied references on Microsoft Excel. Screening of titles and abstracts was conducted with reference to the inclusion/exclusion criteria by KU and JD. After agreeing on the articles eligible for full-text assessment, a full-text review (of 65 articles and dissertations) was conducted independently by two authors, KU and JD (see Figure 1). The two authors had an agreement rate of 92% and resolved discrepancies through discussion. A list of the excluded studies at the full-text assessment stage is presented in Supplementary Appendix B.

### Data extraction

Using a form developed specifically for this study (in Microsoft Excel), data extraction from all articles was conducted independently by two authors, KU and JD. With support from LC, KU and JD met to discuss the findings and resolve any discrepancies. Studies were coded for (1) origin of the study (i.e. the country the work was conducted in); (2) study design (i.e. whether the studies were qualitative, quantitative or mixed methods); (3) sample characteristics (i.e. gender, age, intellectual and communicative abilities, co-occurring diagnoses, living situation, employment status, highest level of education, and race/culture/ethnicity of the participants); (4) study description (i.e. what each study was about); and (5) key outcomes (i.e. what each study found). Studies were also coded for answers to the review questions.

### Quality assessment

Studies were assessed using the Mixed Methods Appraisal Tool (MMAT) (Hong et al., 2018). Within the MMAT, five categories of study design (qualitative, quantitative randomized controlled trials, quantitative non-randomized, quantitative descriptive and mixed methods) are identified, with each category having different criteria. Unlike earlier versions of the MMAT, calculation of a score for each paper is discouraged; instead, a description of how the studies meet MMAT criteria is advised. Overall, the studies in this review tended to meet many/all MMAT criteria, except that the participants tended not to be representative of the target population. Due to the limited research in this area, no studies were omitted after the quality assessment. However, the issue of sample representativeness in research on loneliness in autistic adults is specifically discussed later. The quality assessment was independently conducted by two authors KU and JD (see Supplementary Appendix C for details).

### Data synthesis

A narrative approach was used by the first author KU, with support from LC and the other authors, to synthesize data. This process involved collating key information from every included article that had addressed the review questions. An overview of characteristics of the included studies is included at the beginning of the results.

### Community Involvement

The first author is an autistic researcher. There was no additional involvement of the autistic community in this review.

### Positionality of the authors

Most of the authors have been involved with the autistic and broader autism communities either as a self-advocate KU or as an ally (AR, JD, LC). All authors view autism from a social (as opposed to a medical) model of disability (i.e. acknowledging that disability arises as the consequence of the barriers the society creates for autistic people, rather than viewing autism as a disorder/deficit that needs to be fixed/overcome) (Shakespeare, 2006). One author (BLE) specializes in loneliness research.

## Results

In total, 34 of the 1460 identified studies met all inclusion criteria (see Figure 1). The studies were conducted in the United States (*n* = 15), the United Kingdom (*n* = 7), Australia (*n* = 7), Taiwan (*n* = 3), Hungary (*n* = 1), the Netherlands (*n* = 1), Belgium (*n* = 1) and Denmark (*n* = 1) (note that two studies (Caruana et al., 2021; Chen et al., 2016) included participants from two different countries). Study design included quantitative (*n* = 20), qualitative (*n* = 8) and mixed methods (*n* = 6). The publication year of the included studies ranged from 2007 to 2021, and it appeared that the topic received increased attention in recent years; for example, 17 of the included studies (50%) were published between 2018 and 2021.

In the 34 studies included in this review, 2923 autistic participants were represented. As seen in Table 1, autistic participants were typically more likely to be male rather than female; in young to middle adulthood; of average/above average intellectual and communicative ability; experiencing mood disorders as their most common co-occurring diagnosis; living with parents, family members or caregivers; unemployed rather than employed; highly educated and Caucasian (see Supplementary Appendices D and E for details).

**Table 1.** Autistic participant demographics.^a^

Demographics	Nos. of studies in which the demographic breakdowns were reported	Categories	Value
Gender	29 (*n* = 2234)	Male	*n* = 1172 (52%)
		Female	*n* = 982 (44%)
		Other gender identities^b^	*n* = 68 (3%)
		Not reported	*n* = 12 (1%)
Age (years)	25 (*n* = 2260)	Range	14–80
	22 (*n* = 1688)	Median of the mean	29.6
Intellectual and communicative abilities^c^	8 (*n* = 471)	Please refer to the Supplementary Appendix E for details.	
Co-occurring diagnoses^d^	3 (*n* = 175)	Mood disorders	*n* = 51 (29%)
		Anxiety	*n* = 26 (15%)
		Attention-deficit hyperactivity disorder (ADHD)	*n* = 26 (15%)
Living situation^d^	15 (*n* = 1587)	Living with parents, family members or caregivers	*n* = 843 (53%)
		Living independently (alone, with a partner or with roommates)	*n* = 614 (39%)
		Living in other situations (e.g. supported housing, community home)	*n* = 111 (7%)
Employment status^d^	12 (*n* = 1268)	Unemployed (unable to work, retired, volunteer, living on disability allowance)	*n* = 670 (53%)
		Employed (full-time, part-time, self-employed, student or carer)	*n* = 547 (43%)
Highest level of education^d^	16 (*n* = 1552)	University qualifications or above	*n* = 296 (19%)
		High school level qualifications or below	*n* = 196 (13%)
		Currently in higher education	*n* = 175 (11%)
		A certificate, diploma, associate’s degree or higher vocational education	*n* = 97 (6%)
Race	10 (*n* = 1194)	Caucasian	*n* = 921 (77%)
		Other ethnic groups (e.g. Asian, Black, Hispanic)	*n* = 273 (23%)

a Note: numbers do not add up to total due to rounding/missing data.

b Other gender identities were included in three recent studies: Cage et al. (2018, *n* = 15, 14% of the study sample), Ee et al. (2019, *n* = 10, 5% of the study sample), Hull et al. (2017, *n* = 7, 8% of the study sample), Jackson et al. (2018, *n* = 4, 7% of the study sample) and Levinson (2020, *n* = 32, 26% of the study sample).

c The data are not showed in this category because the measures used to assess the intellectual and communicative abilities are varied in all four studies where the data on this were reported, and additionally, where the data were reported, the measures used to assess only the general descriptions on the intellectual and communicative abilities of their participants were reported in the other four studies.

d A range of other co-occurring conditions were mentioned, and some of which (e.g. anxiety, mood disorders, ADHD; presented in Table 1) were reported more frequently than others (e.g. eating disorders, borderline personality disorder, dyspraxia and dyslexia; see Table 1).

As noted above, most studies met some or all of the MMAT criteria (see Supplementary Appendix C). Common weaknesses identified with the studies included a failure to report the response rate (in quantitative descriptive studies) and a lack of clear descriptions of the target population (in quantitative non-randomized and quantitative descriptive studies).^3^ In addition, most of the included studies failed to represent the diversity of the autistic adult population (e.g. minority ethnic groups were underrepresented).

We considered the design of the studies that contributed to each review question. As such, for each question below, we first delineate whether the results are derived from quantitative, qualitative or mixed method studies. For the mixed methods studies, we clarify whether the results reported in the section were derived from quantitative data, qualitative data or both. For Review Questions 4 and 5, we clarify the design of each study in parentheses.

### Review Question 1: What do we know about autistic adults’ first-hand descriptions of loneliness?

Overall, 5 of the 34 studies (15%) reported autistic adults’ first-hand descriptions of loneliness (Ee et al., 2019; Elmose, 2020; Hickey et al., 2018; R. S. Smith & Sharp, 2013; Van Hees et al., 2015). All five studies were qualitative in design or featured qualitative elements (i.e. in mixed methods studies). Only one article, by Elmose (2020), focused exclusively on loneliness; in the other articles, loneliness was mentioned within a broader focus of investigation (e.g. socialization, diagnosis, sensory experiences, higher education). In four of the five studies (Elmose, 2020; Hickey et al., 2018; Smith & Sharp, 2013; Van Hees et al., 2015), autistic adults’ descriptions of loneliness were elicited using individual or focus group interviews, while Ee et al. (2019) used open-ended surveys.

Elmose (2020) used phenomenological thematic analysis to analyze focus group and individual interview data from 25 autistic adults (18 males, 7 females; 18–71 years of age) who self-reported as autistic. Elmose (2020) reported that autistic adults’ understanding of loneliness was similar to that of non-autistic adults. However, findings also demonstrated that being autistic was perceived to have a major influence on people’s social relationships: ‘Persons with autism have a different perception compared to neurotypical persons. It is evident that this will lead to loneliness’ (P15) (Elmose, 2020, p. 11). Elmose (2020) further reported that discrepancies between desired and actual social relationships caused loneliness in autistic adults. These discrepancies were felt to be caused by several factors, including feeling not understood or misunderstood, creating boundaries that could hinder the possible development of close relationships and masking in an attempt to connect with others. Elmose’s (2020) participants also described their experiences of loneliness: ‘when you are lonely, then it is because you are not able to do anything about it yourself. You do not have the energy. You do not have the tools’ (P1), ‘being locked tightly in a position that you do not wish for’ (P4) (Elmose, 2020, p. 11).

Ee et al. (2019) conducted a mixed methods study using data from a questionnaire-based longitudinal study with 220 autistic adults (86 males, 124 females, 10 other; 25–80 years of age) and 146 non-autistic adults (29 males, 117 females; 25–79 years of age). Quantitative approaches were used to measure loneliness in autistic adults, with qualitative approaches (thematic analysis) used to analyze optional open-ended responses on socialization. Autistic participants in this research emphasized the barriers to, and challenges of, socializing. They also highlighted how the manner in which they experienced loneliness was not the same as being alone: ‘I like being with myself a lot’, ‘I’m alone but not lonely’ (p. 188).

Hickey et al. (2018) thematically analyzed qualitative semi-structured interview data from 13 late-diagnosed autistic adults (10 males, 3 females; 51–71 years of age) who did not have intellectual disabilities and could take part in a verbal interview. Participants reported on their experiences of getting an autism diagnosis, getting support and getting older. One of the three themes identified from these data was longing for connection, which included the sub-theme of isolation and loneliness. It was mentioned that: ‘it’s not to do with not having friends and stuff like that. It’s to do with I just feel that I’m totally isolated in myself’ (Hickey et al., 2018, p. 362). A desire for connection was reported both before and after a diagnosis of autism, and Hickey et al. (2018) concluded that social isolation and loneliness were continual challenges faced by autistic people throughout adulthood.

Smith and Sharp (2013) used modified Grounded Theory (Charmaz, 2006) to analyze semi-structured interview data from nine autistic adults (possibly five men and four women, assumed from their assigned anonymous names), aged 25–49 years. Interviews focused on sensory experiences and were conducted online, through Instant Messenger. Under one of the nine themes identified from these data (‘isolation’), a participant discussed the negative effects of loneliness: ‘it is hell I feel so alone and lonely’ (Smith & Sharp, 2013, p. 902). Helplessness regarding trying to foster connections with others was also described: ‘I don’t think you can stop it (avoiding to go out with friends) or make it go away you just have to accept that’s how it is and learn to live with it’ (Smith & Sharp, 2013, p. 902).

Finally, Van Hees et al. (2015) used principles of Grounded Theory to analyze semi-structured interview data about the experiences of higher education among 23 autistic young adults (17 men, 6 women; 18–25 years of age). Participants’ methods of communication were not reported, but all of them were attending university at the time of the interviews (giving some indication of their cognitive ability). Under the sub-theme of ‘awareness of social problems’ (within one of five themes: ‘exhausting but necessary social contacts’), one participant explained: ‘I’m a lonely person socially. I do not meet many people. I’m lonely’ (Van Hees et al., 2015, p. 1679). The same participant also described their social life, challenging the notion of autistic adults not wanting to socialize: ‘I do not take the initiative. But if there is an offer, I accept it and want to go out’ (Van Hees et al., 2015, p. 1679).

In summary, the results highlighted autistic adults’ desire to have social connections with others, even though social interactions could be challenging. Loneliness was not perceived to be synonymous with being alone, but was a negative and persistent feeling for the autistic adults.

### Review Question 2: how is loneliness in autistic adults measured?

Overall, 22 of the 34 (65%) studies used self-report questionnaires to measure loneliness in autistic adults (with autistic sample sizes ranging from 17 to 220) (see Supplementary Appendix F). Data that contributed to this review question were all quantitative (from both quantitative and mixed methods studies). Eight different loneliness questionnaires were used across the studies. Four questionnaires were different versions of the UCLA Loneliness Scale: the UCLA Loneliness Scale Short Form (ULS-8) (Hays & DiMatteo, 1987) (used in Ee et al., 2019; Hedley, Uljarević, Foley, et al., 2018; Lin & Huang, 2019; Mazurek, 2013, 2014; Sundberg, 2018; Syu & Lin, 2018), the UCLA Loneliness Scale Version 3 (Russell, 1996) (used in Brooks, 2014; Hedley, Uljarević, Wilmot, et al., 2018; Hillier et al., 2018; Jantz, 2011; Russell, 2020), the Revised UCLA Loneliness Scale (Russell et al., 1980) (used in Caruana et al., 2021; Levinson, 2020) and the 3-item UCLA Loneliness Scale (Hughes et al., 2004) (used in Jackson et al., 2018). One further study, by van der Aa et al. (2016), used six items based on the Revised UCLA Loneliness Scale (Russell et al., 1980) to measure loneliness in autistic adults, yet further information about the rationale for selecting these particular items could not be gathered from the authors. The other questionnaires used were the Social and Emotional Loneliness Scale for Adults (SELSA) (DiTommaso & Spinner, 1993) (used in Bourdeau, 2020; Gantman et al., 2012; McVey et al., 2016; Merkler, 2007; Schiltz et al., 2020), the Loneliness in Context Questionnaire (LiCQ) (Asher & Weeks, 2014) (used in Han et al., 2019) and Isolation and Affect measure (developed and used in Merkler, 2007). Importantly, the UCLA Loneliness Scales, SELSA and LiCQ were developed to measure loneliness in the general population and the validity of these measures for the autistic population has not yet been established. One study (McVey et al., 2016) used the SELSA (DiTommaso & Spinner, 1993) and reported the internal consistency (0.71) within their autistic sample (see Supplementary Appendix F).

In just one study, a measure of loneliness was specifically developed for autistic adults. Merkler (2007) created an Isolation and Affect measure to distinguish isolation and affect as two distinct components of loneliness among autistic adults and neurotypical university students. This scale was based on the Peer Network and Dyadic Loneliness Scale (PNDLS) (Hoza et al., 2000) designed to assess loneliness in children within the context of both social peer networks and dyadic relationships. Merkler (2007) modified the wording of items to be applicable to adult participants and, through a confirmatory factor analysis and by correlating the measure with other similar measures (e.g. the SELSA), the Isolation and Affect measure was shown to be valid in their sample.

Seven studies (Brooks, 2014; Ee et al., 2019; Han et al., 2019; Levinson, 2020; Lin & Huang, 2019; Russell, 2020; Sundberg, 2018) included comparison groups in their studies and reported loneliness scores for both autistic and non-autistic adults (indicated with asterisks in Supplementary Appendix F). In all seven studies, the autistic group had higher levels of loneliness than the non-autistic comparison group. Two of these studies included additional comparison groups of non-autistic adults who had other diagnoses. In one study, Russell (2020) reported that non-autistic adults who suffered from insomnia reported loneliness that was equivalent to that of autistic adults. In the other study, Han et al. (2019) reported that non-autistic adults who were clinically depressed at the time of the study reported higher levels of loneliness than autistic and non-autistic adults who were not clinically depressed (Han et al., 2019). See Table 2 for details.

**Table 2.** Comparison groups and matching procedure.

Study	Comparison group	Matching procedure
Brooks (2014)	Typically developing (TD) participants	TD participants were recruited through ResearchMatch.org, an online database to match participants for research. TD participants with scores greater than 25 on the Autism Spectrum Quotient (AQ) (Baron-Cohen et al., 2001) were excluded.
Ee et al. (2019)	Non-autistic adults	NR
Han et al. (2019)	TD-depressed, TD-controls	The ADOS-2 Module 4 was administered for all participants in the ASD group and any participants who exceeded the clinical cut-offs on social responsiveness scale (SRS-2) (Constantino & Gruber, 2012) or AQ (Baron-Cohen et al., 2001). Using the structured clinical interview for *DSM* disorders (SCID-5) (First et al., 2016) depression module and the mini international neuropsychiatric interview (MINI 5.0) (Sheehan et al., 1998), all participants were assessed for emotional health history.
Levinson (2020)	Neurotypical young adults	NR
Lin & Huang (2019)	Neurotypical adults	The exclusion criteria for neurotypical adults included having ‘(a) any physical disabilities, visual impairment, hearing impairment or developmental disabilities; and (b) attending special education schools or classes’ (Lin & Huang, 2019, p. 3).
N. C. C. Russell (2020)	Typically developing individuals (NT), adults show showed at least subthreshold insomnia symptoms (INS)	Autistic adults’ autism diagnoses were confirmed using the Autism Diagnostic Observation Schedule – Second Edition (Lord et al., 2012). INS group included those who scored 10 and above on the Insomnia Severity Index (ISI; Bastien et al., 2001). NT group did not have ‘history of severe head trauma or neurological condition’ (N. C. C. Russell, 2020, p. 24) and scored 7 or below on the ISI.
Sundberg (2018)	Control	NR

NR: not reported. Terminology to describe autism corresponds to the exact terms in each article and it is not the intention of the review.

### Review Question 3: what dimensions of loneliness (intimate, relational or collective) have been reported in research on autistic adults?

We categorized every included study (quantitative: *n* = 20, qualitative: *n* = 8 and mixed methods: *n* = 6) into one of the three dimensions of loneliness: intimate, relational or collective (see Table 3). In the mixed methods studies, both quantitative and qualitative aspects of the data contributed to this review question. Next, we report on the dimension(s) of loneliness that were evident from the context of the studies (e.g. if a finding was reported on friendship, this was categorized as relational loneliness) and/or we report on the dimensions of loneliness that the measure(s) used within the study appeared to assess.

**Table 3.** Dimensions of loneliness.

Study	Loneliness measures	Study design	Dimensions of loneliness		
			Relational	Intimate	Collective
Ashbaugh et al. (2017)	NA	Quantitative	✓		✓
Baldwin & Costley (2016)	NA	Mixed methods	✓	✓	
Bourdeau (2020)	SELSA	Quantitative	✓	✓	
Brooks (2014)	The UCLA Loneliness Scale, Version3	Quantitative	✓		
Caruana et al. (2021)	Revised UCLA Loneliness Scale	Quantitative	✓		
Chen et al. (2016)	NA	Quantitative	✓	✓	
Ee et al. (2019)	ULS-8	Mixed methods	✓		
Elmose (2020)	NA	Qualitative	✓		✓
Gantman et al. (2012)	SELSA	Quantitative	✓	✓	
Han et al. (2019)	LiCQ	Quantitative	✓		
Hedley, Uljarević, Foley, et al. (2018)	The UCLA Loneliness Scale, Version3	Quantitative	✓		
Hedley, Uljarević, Wilmot, et al. (2018)	ULS-8	Quantitative	✓		
Hickey et al. (2018)	NA	Qualitative	✓	✓	
Hillier et al. (2018)	The UCLA Loneliness Scale, Version3	Mixed methods	✓		
Hull et al. (2017)	NA	Qualitative	✓	✓	✓
Hwang et al. (2017)	NA	Qualitative	✓		✓
Jackson et al. (2018)	3-item UCLA Loneliness Scale	Quantitative	✓	✓	
Jantz (2011)	The UCLA Loneliness Scale, Version3	Mixed methods	✓		✓
Levinson (2020)	Revised UCLA Loneliness Scale	Quantitative	✓		
Lin & Huang (2019)	ULS-8	Quantitative	✓		
Mazurek (2013)	ULS-8	Mixed methods	✓		
Mazurek (2014)	ULS-8	Quantitative	✓		
McVey et al. (2016)	SELSA	Quantitative	✓	✓	
Merkler (2007)	SELSA and Isolation and Affect measure	Quantitative	✓	✓	
Milton & Sims (2016)	NA	Qualitative	✓		✓
Orsmond et al. (2013)	NA	Quantitative	✓		
Russell (2020)	The UCLA Loneliness Scale, Version3	Quantitative	✓		
Schiltz et al. (2020)	SELSA	Quantitative	✓	✓	
Smith & Sharp (2013)	NA	Qualitative	✓		
Southby & Robinson (2018)	NA	Qualitative	✓		
Sundberg (2018)	ULS-8	Quantitative	✓		
Syu & Lin (2018)	ULS-8	Quantitative	✓		
van der Aa et al. (2016)	Loneliness Scale based on the Revised UCLA loneliness scale	Mixed methods	✓		
Van Hees et al. (2015)	NA	Qualitative	✓		

SELSA: Social and Emotional Loneliness Scale for Adults.

Relational loneliness (i.e. peer relationships) was researched most, featuring in every included study; quantitative (*n* = 20), qualitative (*n* = 8) and mixed methods (*n* = 6). Intimate loneliness (i.e. romantic relationships) was explored in 10 studies (Baldwin & Costley, 2016; Bourdeau, 2020; Chen et al., 2016; Gantman et al., 2012; Hickey et al., 2018; Hull et al., 2017; Jackson et al., 2018; McVey et al., 2016; Merkler, 2007; Schiltz et al., 2020), comprising quantitative (*n* = 7), qualitative (*n* = 2) and mixed methods (*n* = 1) studies. Collective loneliness (i.e. a sense of belonging in society) was explored in 6 studies (Ashbaugh et al., 2017; Elmose, 2020; Hull et al., 2017; Hwang et al., 2017; Jantz, 2011; Milton & Sims, 2016); quantitative (*n* = 1), qualitative (*n* = 4) and mixed methods (*n* = 1) studies.

### Review Question 4: what factors are associated with increased loneliness in autistic adults?

Factors positively associated with social isolation and/or loneliness in autistic adults were reported in 18 of the 34 studies (53%), including quantitative (*n* = 10), qualitative (*n* = 5) and mixed (*n* = 3) methods studies. The factors identified are presented in the order of frequency (from most to least commonly reported). Note that most of the quantitative studies tended to be correlational in nature (as opposed to causal).

#### Autistic characteristics

In total, 13 studies (9 quantitative, 1 qualitative and 3 mixed methods) identified autistic characteristics as a factor positively associated with loneliness among autistic adults. Of those, 10 of the 13 studies (Brooks, 2014; Caruana et al., 2021; Ee et al., 2019; Hedley, Uljarević, Foley, et al., 2018; Hedley, Uljarević, Wilmot, et al., 2018; Jantz, 2011; Mazurek, 2014; Schiltz et al., 2020; Syu & Lin, 2018; van der Aa et al., 2016) found an association between loneliness and scores on variations of the Autism Quotient (AQ) (Baron-Cohen et al., 2001). Meanwhile, 2 of the 13 studies (Chen et al., 2016; Han et al., 2019) identified an association between loneliness and scores on the Social Responsiveness Scale Second Edition (SRS-2) (Constantino & Gruber, 2012; see Table 4 for statistics reported in the quantitative studies.). In one of the qualitative studies (Elmose, 2020), autistic adults reported that being autistic was linked to their experiences of loneliness and underlying social experiences.

**Table 4.** Statistics on the association between autistic characteristics and loneliness.

Study	Analysis	Statistics
Schiltz et al. (2020)	Pearson’s correlations	(*r* = 0.41–0.49, *p* < 0.01) *Social (*r* = 0.49, *p* < 0.01) and family (*r* = 0.41, *p* < 0.01) subscales of the SELSA
Caruana et al. (2021)	Spearman’s correlation	(Spearman ρ = 0.278, *p* < 0.001) for autistic characteristics and anthropomorphism, (Spearman ρ = 0.242, *p* = 0.024) for anthropomorphism and loneliness
Brooks (2014)	Pearson’s correlations	(*r* = 0.350, *p* = 0.05)
Hedley, Uljarević, Wilmot, et al. (2018)	Pearson’s correlations	(*r* = 0.331, *p* < 0.01)
Hedley, Uljarević, Foley, et al. (2018)	Pearson’s correlations	(*r* = 0.232, *p* < 0.05)
Jantz (2011)	Pearson’s correlations	(*r* = 0.334, *p* ⩽ 0.05)
Mazurek (2014)	One-way ANOVA	(β = 0.28, *p* = 0.004)
Syu & Lin (2018)	One-way ANOVA	(β = 0.345, *p* = 0.004)
Ee et al. (2019)	Regression	(β = 0.104, *p* = 0.003)
van der Aa et al. (2016)	Regression	(β = −0.54, *p* < 0.001) *The authors conducted a regression analysis using AQ as a measure of autistic characteristics as continuous variables afford a ‘statistically more robust result’ (p. 21) than dichotomous variables, such as diagnostic status. Thus, it should be noted that there was some overlap in the samples in the regression, with some autistic people not scoring above the cut-off on the AQ to be considered autistic and vice versa.
Chen et al. (2016)	Multilevel linear analyses	(β = −0.10, *p* < 0.05) *Chen et al. (2016) reported that increased severity on the SRS moderated the relationships between loneliness and interactions with others at work/school.
Han et al. (2019)	Linear regressions	Capacity for social pleasure (*t*(96) = 2.52, *p* = 0.01), capacity for non-social pleasure (*t*(95) = 2.60, *p* = 0.01) *Han et al. (2019) found that higher levels of autistic traits predicted loneliness in autistic adults. This association, however, was moderated by anhedonia, loss of pleasure.

SELSA: Social and Emotional Loneliness Scale for Adults; ANOVA: analysis of variance; AQ: Autism Quotient; SRS: Social Responsiveness Scale.

#### Heightened anxiety

Four studies (three quantitative, one mixed methods) reported heightened anxiety as a factor positively associated with loneliness in autistic adults. Schiltz et al. (2020) reported a positive correlation between social (*r* = 0.52–0.59^4^, *p* < 0.01) and emotional (*r* = 0.40–0.47, *p* < 0.01) loneliness subscales on the SELSA (DiTommaso & Spinner, 1993) and social anxiety in autistic adults. Mazurek (2014) reported that loneliness and social isolation were positively correlated with anxiety (*r* = 0.34, *p* = 0.001) (and depression, low self-esteem and low quality of life) in autistic adults. Furthermore, Chen et al. (2016) found that greater severity of autistic characteristics on the SRS was associated with more ‘in-the-moment’ anxiety (p. 1411) (β = −0.07, *p* < 0.01). The researchers added that anxiety might make autistic adults more self-aware of social limitations and perceived social incompetence, leading to feelings of loneliness. Finally, Ee et al. (2019) found that autistic adults with higher scores on the Severity Measure for Generalized Anxiety Disorder-Adult (Craske et al., 2013) were lonelier than those with lower scores (β = 0.216, *p* < 0.001).

#### Depression and suicidal ideation

Four studies (three quantitative and one mixed methods) reported depression and suicidal ideation as factors positively associated with loneliness in autistic adults. Mazurek (2014) found loneliness was positively associated with depression (*r* = 0.48, *p* < 0.001) in autistic adults. Also, Schiltz et al. (2020) reported that the social (*r* = 0.44, *p* < 0.01) and emotional (*r* = 0.72, *p* < 0.01) subscales on the SELSA were positively associated with depression in autistic adults. Furthermore, Jackson et al. (2018) found that lifetime suicidal behaviours were positively associated with loneliness in autistic adults (*r*_s_(53) = 0.36, *p* < 0.01). Finally, Ee et al. (2019) identified that autistic adults with higher scores on Patient Health Questionnaire-9 (Kroenke et al., 2001) were lonelier than autistic adults with lower scores (β = 0.30, *p* < 0.001).

#### Negative experiences and learned helplessness

Three studies (two qualitative and one mixed methods) identified negative experiences and learned helplessness as factors positively associated with loneliness among autistic adults. Likewise, in Ee et al.’s (2019) mixed methods study, participants explained that their past experiences impacted their desire for socialization, with negative experiences, such as bullying leading them to avoid socialization: ‘people have been so cruel to me, I don’t socialize ever anymore’ (p. 189). Milton and Sims (2016) conducted a thematic analysis of the narratives of autistic adults in an autism-related magazine. They reported that loneliness in autistic adults was linked to negative experiences in social situations (i.e. bullying) that arose as a result of having an ‘othered’ identity. Smith and Sharp (2013) conducted semi-structured interviews on sensory experiences and their qualitative analysis suggested that autistic adults experienced rejection from others due to their unique sensory experiences and that such experience of rejection could lead to loneliness.

#### Lack of autism understanding and acceptance from others

Three qualitative studies reported others’ lack of autism understanding and acceptance as a factor positively associated with loneliness in autistic adults. While Milton and Sims (2016) did not use the term loneliness, narratives of autistic adults in their research described how a lack of understanding from others caused them to feel ‘othered’ and less connected. In turn, participants sought to connect with people who understood them:

I cannot talk about my real experience of life to most people, because they wouldn’t understand or be interested. That makes me feel, as the saying goes, ‘lonely in a room full of people’ and I’m fed up with it. I would like to talk to caring, intelligent, honest people who understand Asperger’s well and with whom I can talk openly. (Daniel, Pen Pal 95, issue 68, 7, Milton & Sims, 2016, p. 529)

Elmose (2020, p. 11) reported that autistic adult participants in her study felt ‘positioned by others’ and misunderstood by those around them. Such feelings of misunderstanding were suggested to be associated with loneliness, as one participant explained: ‘it is probably in those situations I feel lonely’ (P19, Elmose, 2020, p. 14). Finally, Hwang et al. (2017) reported that a lack of autism awareness and understanding caused negative social experiences for autistic adults, including bullying and social isolation. A mother of an autistic adult in this study described the way in which a lack of autism understanding and acceptance made her grown-up child feel lonely:

People ignore him a lot . . . and they don’t talk to him and they do avoid him and ignore him and given that he struggles with eye contact and then other people avoid eye contact with him . . . It affects him more than we realise. So I guess that’s awareness . . . They get shunned and ostracised a lot, you know. A lot of loneliness . . . we’re all intolerant aren’t we. Intolerant. Ignorant. (Hwang et al., 2017, p. 2041)

#### Sensory avoidance

Two studies (one quantitative and one qualitative) identified sensory avoidance as a factor positively associated with loneliness among autistic adults. Smith and Sharp (2013) reported that sensory avoidance due to sensory stressful environments render autistic adults socially isolated, which could lead to loneliness. Furthermore, Syu and Lin (2018) reported that autistic adults with higher scores on sensory avoidance in the Chinese version of the Adult Sensory Profile (Tseng & Chen, 2009) showed higher levels of loneliness (β = 413, *p* = 0.009).

#### Camouflaging

Camouflaging refers to ‘coping skills, strategies, and techniques that function to “mask” features of [autism] during social situations’ (Hull et al., 2017, p. 2523). Two qualitative studies reported camouflaging as a factor positively associated with loneliness in autistic adults. Hull et al. (2017) argued that camouflaging makes it easier to make connections with others because, in the words of an autistic participant, ‘connections have to be made initially on neurotypical terms’ (Hull et al., 2017, p. 2523). Hull et al. (2017) further explained that relationships formed when camouflaging may be perceived as false by some autistic adults, which can leave them with the feelings of loneliness (Hull et al., 2017). In addition, Elmose’s (2020) qualitative study also reported that autistic adults engaged in camouflaging and this was linked to their experiences of loneliness.

#### Unemployment

Just one mixed methods study reported unemployment as a factor positively associated with loneliness in autistic adults. Ee et al. (2019) included autistic and non-autistic adults in their study and, using regression analyses, reported that unemployment was associated with increased loneliness only among autistic adults (β = 1.45, *p* = 0.045).

### Review Question 5: what factors are associated with decreased loneliness in autistic adults?

The factors negatively associated with loneliness in autistic adults were reported in 18 of the 34 studies (53%): a combination of quantitative (*n* = 8), qualitative (*n* = 6) and mixed methods (*n* = 4) studies. The reported factors are presented in the order of frequency of reports (from most reported to least reported). Note that most of the quantitative studies tended to be correlational in nature (as opposed to causal).

#### Having relationships

Overall, 16 studies (7 quantitative, 6 qualitative and 3 mixed methods) reported having relationships as a factor negatively associated with loneliness in autistic adults. Of those, 10 of the 16 studies (Bourdeau, 2020; Brooks, 2014; Hedley, Uljarević, Foley, et al., 2018; Jackson et al., 2018; Jantz, 2011; Mazurek, 2013, 2014; Schiltz et al., 2020) reported statistics on the association between having relationships (i.e. friendships, social participation/contacts in general) and loneliness (see Table 5). In an evaluation of a social skills training for autistic young adults, Gantman et al. (2012) found that participants experienced a decrease in self-reported loneliness following the training. The authors suggested that the development of friendships during the training might explain the decline in participants’ loneliness. Similarly, Hillier et al. (2018) investigated the impacts of a social intervention programme for autistic adults on their loneliness, self-esteem and mental health and suggested that the observed reduction in loneliness was because autistic adults were able to develop relationships with peers.

**Table 5.** Statistics for the association between having relationships and loneliness.

Study	Analysis	Statistics
Bourdeau (2020)	rANOVA	Wilks’ Lambda = 0.29, *F*(1, 36) = 89.97, *p* = 0.71, ES = 0.714
Mazurek (2014)	One-way ANOVA	(β = −0.22, *p* = 0.02) for close friendship and loneliness
Brooks (2014)	Pearson’s correlations	*r* = −0.467, *p* < 0.001 for friendship quality and loneliness
Jackson et al. (2018)	Regression	(*r*_s_(54) = −0.52, *p* < 0.001) for the number of close friends and loneliness, (*r*_s_(54) = −0.61, *p* < 0.001) for satisfaction with the number of close friends
Jantz (2011)	Pearson’s correlations	(*r* = −0.492, *p* ⩽ 0.05) for the number of close friends and loneliness, (*r* = −0.398, *p* ⩽ 0.05) for the number of social engagements and loneliness
Mazurek (2013)	One-way ANOVA	β = −0.30, *p* = 0.003 for the number of friends and loneliness
Schiltz et al. (2020)	Pearson’s correlations	*r* = −0.53, *p* < 0.01 for social loneliness, *r* = −0.27, *p* < 0.05 for family loneliness on SELSA
Hedley, Uljarević, Foley, et al. (2018)	Regression	(β = 0.43, *p* < 0.001) for the number of social supports and loneliness, (β = −0.47, *p* < 0.001) for satisfaction with social support and loneliness

SELSA: Social and Emotional Loneliness Scale for Adults; ANOVA: analysis of variance

Three qualitative studies (Elmose, 2020; Milton & Sims, 2016; Southby & Robinson, 2018) indicated that having relationships, particularly through shared interests, may alleviate loneliness. For example, Southby and Robinson (2018) proposed that participants who attended the Leeds Autism AIM (advocacy, information and mentoring) service felt less socially isolated as they had an opportunity to engage with ‘likeminded people’ (p. 514). Another qualitative study showed that married autistic adults felt less lonely than those who were not married (Hickey et al., 2018). Explaining their findings, Hickey et al. (2018) proposed that having one close relationship provided some sense of connection and therefore reduced loneliness. Van Hees et al. (2015) used interviews to explore autistic adults’ experiences of higher education and found that a scarcity of relationships was associated with higher levels of loneliness, while supportive relationships could alleviate feelings of loneliness. Finally, Smith and Sharp (2013) explored autistic adults’ sensory experiences and reported that having positive relationships, such as with family or friends, could make autistic adults less vulnerable to social isolation and loneliness.

#### Participation in social skill interventions and/or experiencing fewer difficulties with social skills

Two studies (one quantitative and one mixed methods) reported that participation in social skills interventions and/or experiencing fewer difficulties with social skills was a factor negatively associated with loneliness in autistic adults. Gantman et al. (2012) adapted and tested the effectiveness of a social skills intervention for autistic adolescents, the Program for the Education and Enrichment of Relational Skills (PEERS, Laugeson & Frankel, 2011), with autistic young adults. They found that PEERS social skills training was associated with reduced loneliness. However, it is worth noting that McVey et al. (2016) replicated this work and did not find PEERS to be associated with reduced loneliness in autistic young adults (*F*(1, 16) = 4.73, *p* < 0.05). Using multiple regression, Ee et al. (2019) explored the factors that were associated with loneliness in autistic and non-autistic adults. They found that higher scores on the subscale of social skills on the AQ-Short (where higher scores indicate more autistic characteristics) (Hoekstra et al., 2011) were associated with decreased loneliness in autistic adults (β = 0.446, *p* < 0.001). The AQ-Short has 28 items with two major domains: social behavioural difficulties and fascination for numbers/patterns. The social behavioural difficulties domain contains the subdomain of social skills (e.g. ‘I find it hard to make new friends’, ‘I would rather go to a library than to a party’).

#### Positive views and acceptance of oneself

Three studies (one quantitative, one qualitative and one mixed methods) reported self-esteem and acceptance as factors negatively associated with loneliness in autistic adults. Mazurek (2014) reported that loneliness was negatively correlated with self-esteem (*r* = −0.38, *p* < 0.001) and life satisfaction (*r* = −0.46, *p* < 0.001) in autistic adults. Acceptance of autistic identity was also associated with lower feelings of isolation, according to a study involving a thematic analysis of issues of the magazine *Asperger United* (AU)^5^ (Milton & Sims, 2016). From the quantitative data in Ee et al.’s (2019) mixed methods study, it was found that self-efficacy was associated with less loneliness in autistic adults (β = −1.291, *p* < 0.001).

#### Female gender

One mixed methods study (Ee et al., 2019) reported gender as a factor negatively associated with loneliness in autistic adults. In this study, being female was associated with decreased loneliness (β = −2.62, *p* = 0.004).

#### Time spent engaging in activities

Sundberg (2018) examined how online gaming affects friendships and loneliness in autistic teenagers and adults, finding that autistic individuals who played online games less than 1 h per day experienced significantly less loneliness than those who played 2–3 h (*p* = 0.049) or 3–5 h per day (*p* = 0.01).

## Discussion

This is the first systematic review to examine loneliness in autistic adults. A key finding from this review was that research on this topic is in its infancy: few studies have examined loneliness in autistic adults exclusively, with existing studies tending to examine loneliness as part of broader research investigations; no studies have reported on the characteristics of autistic adults who are lonely versus those who are not; few studies have included comparison groups of non-autistic adults; most studies only report quantitative data on loneliness with less information provided on the qualitative descriptions of loneliness perceived by autistic adults; and there is a lack of diversity of research participants in work on this topic regarding age, gender, ability levels and race/culture/ethnicity. Despite these gaps in the literature, the work included in this review has provided several important contributions to our understanding of loneliness in autistic adults. For example, the results demonstrated that autistic adults do desire connection and do experience loneliness; autistic adults report higher scores on measures of loneliness than their non-autistic peers; and some factors associated with loneliness are common among autistic and non-autistic groups, while others appear unique to the autistic population. These conclusions are based on both qualitative and quantitative work. Next, we reflect on the strength and nature of the existing literature on loneliness among autistic adults, using these findings to suggest both avenues for future research and implications for practice.

Research reporting on autistic adults’ first-hand experiences of loneliness highlighted autistic people’s desire for social connections, despite experiencing difficulties in social situations. While loneliness was negatively perceived, and sometimes viewed as an inevitable consequence of challenges in social situations, autistic adults expressed a desire for a sense of connection. The social motivation theory of autism (Chevallier et al., 2012) suggests that autistic children are less interested in social involvement than non-autistic children, and that such indifference eventually leads to poorer development in social communication and interaction. Yet, existing research shows that autistic children *do* desire friendships (Bauminger & Kasari, 2000; Calder et al., 2013) and that this desire extends into adulthood (Gillespie-Lynch et al., 2017). Consistent with these findings, studies included in this review note how autistic people experience loneliness and long for connection and belonging in the same way that non-autistic people do. However, the way that autistic adults experience ‘the world of people’ (Grandin & Scariano, 1986, p. 19) appears to be different. Despite a desire for connection, autistic adults may be less likely to have opportunities for such connection. For example, autistic adults are no longer in the mandatory social setting of school and are less likely to be in employment to forge social connections with colleagues. It is, therefore, essential to consider how to alleviate feelings of loneliness among autistic adults.

Results from this review demonstrate the value of autistic adults having social relationships to alleviate loneliness. For example, the autistic participants in Elmose’s (2020) research reported that factors, such as sharing interests, and a sense of safety, recognition and acceptance, made it easier for them to socially interact with others. Likewise, several studies demonstrated how autistic adults often found value in social relationships with other autistic people (Elmose, 2020; Milton & Sims, 2016; Southby & Robinson, 2018). These findings link with recent research outside the field of loneliness, which has shown that autistic/autistic interactions are perceived as easier and more comfortable than autistic/non-autistic interactions (Crompton, Hallett, et al., 2020; Crompton, Ropar, et al., 2020), and that interacting with other autistic friends and family members provides autistic adults with a sense of belonging (Crompton, Hallett, et al., 2020). This supports growing calls for autistic peer support, for which initial evaluations have yielded positive results (e.g. Crane et al., 2020). These findings do not, however, imply that autistic adults should *only* forge social connections with other autistic adults. Indeed, characteristics of successful autistic/non-autistic relationships have been documented (Smith et al., 2021).

The question then arises of how to measure loneliness in autistic adults. Standardized measures of loneliness were used in ~65% studies included in the review. The results of these studies consistently showed that autistic adults had higher levels of loneliness than their non-autistic peers. However, this finding should be interpreted with caution since (despite the high quality of the studies, as rated on the MMAT) most loneliness measures used in these research studies have not been specifically designed for, or validated with, autistic people. For example, on the widely used UCLA Loneliness Scale (Russell, 1996), respondents are asked ‘how often do you feel close to people?’, which autistic adults may interpret literally (Mason et al., 2019). Outside of loneliness research, there have been attempts to develop autism-specific measures of suicidality (Cassidy et al., 2018) and quality of life (McConachie et al., 2018), partly due to concerns that existing measure of suicidal ideation or quality of life index factors inextricably linked to being autistic. Importantly, no studies have fully examined the validity of existing measures of loneliness for the autistic population. While Merkler (2007) developed a loneliness measure for autistic adults, more in-depth consultation and collaboration with the autistic community during such a process would be beneficial; akin to efforts that have been made in the field of quality of life and suicidality (e.g. Cassidy et al., 2018; McConachie et al., 2018). Future work should also establish how best to measure loneliness in autistic people by identifying whether autism-specific measures of loneliness are needed, or whether existing tools adequately capture autistic experiences of loneliness using participatory research framework (as per Nicolaidis et al., 2020).

The findings of this review highlighted how some factors associated with loneliness appear similar among autistic and non-autistic adults. For example, loneliness has been linked to poorer mental health (e.g. depression, suicidality) among both autistic and non-autistic adults. Existing studies have not, however, examined whether there are autism-specific pathways to these outcomes. In a prominent model of loneliness, Cacioppo and Hawkley (2009) describe a self-reinforcing loop where loneliness leads to hypervigilance for social threats and a bias towards negative social experiences. This, in turn, leads to people experiencing negative social events that confirm their negative social expectations, resulting in further negative social interactions and enhanced loneliness. The results of this review are broadly consistent with this model. For example, characteristics of autism may render autistic adults to be hypervigilant to social threats, resulting in camouflaging. If unsuccessful, this may exacerbate their negative social experiences. Furthermore, heightened anxiety as a driver of loneliness, as found in autistic people (Chen et al., 2016; Mazurek, 2014), has been previously reported in the general population (Caplan, 2007; Mazurek, 2014). Yet, it should be noted that causal interpretation of research on the potential causes of loneliness included in this review was limited as most of the studies used correlational data rather than predictive modelling. Investigating shared/different mechanisms underpinning loneliness in autistic and non-autistic adults more rigorously is an important avenue for further research.

Once the mechanisms underpinning loneliness in autistic adults have been established, it is important to determine how autistic loneliness could be overcome. Existing work in this area is limited, with quantitative studies included in this review largely focusing on correlation as opposed to causation. In terms of developing this work further, one option is to address the internal, predisposing factors that render autistic people vulnerable to loneliness, such as difficulties with social skills. Indeed, our review suggests that some evidence exists for the association between social skills training and decreases in loneliness (Gantman et al., 2012). However, our findings also suggest that trying to ‘fit in’ with the non-autistic population (e.g. through masking) can lead to increases in loneliness. As such, we do not advocate for interventions that encourage autistic individuals to conform to non-autistic people’s social norms. Instead, we encourage autistic people to cultivate positive views and acceptance of themselves. Indeed, self-acceptance was reported to be associated with decreased loneliness in this review (Milton & Sims, 2016).

Alternatively, one could address the external, contributory factors that lead to social isolation and feelings of loneliness among autistic people, such as others’ negative views of autistic differences. Milton’s (2012) double-empathy theory explains how autistic people often struggle to empathize with non-autistic people, but equally the converse is also true. Applying this theoretical framework to loneliness research, this could explain a vicious cycle of negative social experiences for autistic adults, which may render them more vulnerable to loneliness. Similarly, findings from this review indicate that the avoidance of stressful sensory experiences, common in environments set up for the non-autistic norm, may contribute to loneliness in autistic adults. Overall, further work should investigate ways to overcome loneliness in autistic people from both directions: examining what autistic adults can do to overcome feelings of loneliness but also focusing on what non-autistic people and society in general can do to be more accepting and inclusive of autistic differences.

Finally, it is important to reflect on the importance of autistic voice in determining priorities for future research. In the current review, there were both similarities and differences between the findings from quantitative and qualitative research studies. However, it was notable that, collective loneliness was only reported in 5% of the quantitative studies (1 out of 20) compared to 50% of the qualitative studies (4 out of 8). Collective loneliness was also reported in the qualitative data from one mixed methods study (Jantz, 2011). Such differences between the focus of quantitative and qualitative studies suggest a potential discrepancy between the loneliness research priorities of autism researchers and autistic adults. It will be critical for future research to be guided by autistic adults’ research priorities on this topic.

### Limitations

Here, it is important to address the limitations of the studies included in the current review and the limitations of the review itself. Most studies included in this review focused on autistic adults in early to middle adulthood, despite loneliness having a huge impact on autistic people’s quality of life as they age. Likewise, studies tended to focus on adults who had average/above average intellectual and communicative abilities, despite difficulties with speech and cognition increasing the likelihood of social isolation in young autistic adults (Ashbaugh et al., 2017; Brooks, 2014; Chen et al., 2016; Hickey et al., 2018; Merkler, 2007; Syu & Lin, 2018). Russell et al. (2019) recently reported that more than 90% of autistic participants in research studies do not have co-occurring intellectual disabilities. As such, additional work on experiences of loneliness in this group is crucial.

Research studies featured in this review often included a rather narrow definition of loneliness. Specifically, there was a dearth of literature on collective loneliness in comparison with relational and intimate loneliness. It is also noteworthy that little research has been on autistic adults’ relationships with non-human agents with just one included study investigating such relationships in association to loneliness (Caruana et al., 2021). A final limitation to note is that many of the studies featured in this review appeared to assume that loneliness and social isolation were synonymous experiences. For example, many studies used the two terms interchangeably or used level of isolation as a proxy for loneliness. However, qualitative experiences of loneliness and social isolation are likely to differ (Holt-Lunstad et al., 2015; Wang et al., 2017; Zavaleta & Samuel, 2014). It will be important for future research on loneliness in autistic adults to distinguish loneliness from social isolation.

In addition to the limitations of the studies included in this review, there are limitations associated with the review itself. First, only English language articles were included. Second, an examination of the broader context of loneliness (e.g. poor social economic status or housing) was beyond the scope of this review, but is an important consideration for future work. Third, as the studies included in this review largely examined the factors associated with loneliness, as opposed to causal factors underpinning loneliness, this review cannot draw firm conclusions on what causes loneliness, but only on the factors potentially associated with loneliness in this population. Fourth, search terms in this review intentionally focused on loneliness and social isolation, however, including more search terms, such as social network and relationships, might have generated broader results (e.g. Ma et al., 2020). Fifth, the majority of the included studies used loneliness measures developed for the general population and, until their validity and reliability has been established in autistic adults, the results need to be treated with caution (as they might have under-/overestimated loneliness in autistic adults). Sixth, due to the lack of existing work on causation, some of the associations reported in Review Questions 4 and 5 are speculative and require further research before we can be confident of these associations. Finally, as the first review on this topic, we intentionally included broad review questions. Our search strategy for this broad field may therefore not have been fully comprehensive and we were only able to conduct a narrative synthesis of included studies.

### Future research

The current review has highlighted several important directions for future research. First, given that most existing loneliness measures were designed for the general population and not specifically for autistic adults, future research should investigate if, and how accurately, existing loneliness measures capture loneliness in autistic adults. Second, existing quantitative studies on loneliness in autistic adults tended to be correlational in nature. Future research will benefit from exploring the pathways to loneliness in autistic adults, determining if/how they differ to those of non-autistic adults. Third, it is salient to note that the factors impacting loneliness in autistic adults were reported to be both internal and external to autistic adults. Thus, future research needs to investigate both what autistic adults can do to alleviate loneliness and what society in general can do to overcome loneliness in autistic adults. Fourth, the broad overlaps, but also some discrepancies, between the results of quantitative and qualitative research suggest the need to investigate autistic adults’ research priorities on loneliness and to use them to guide subsequent lines of inquiry. Finally, while the quality of the studies as assessed using the MMAT was fairly high, many studies shared key limitations, notably around the representativeness of the samples. These limitations are not unique to research on autistic loneliness (e.g. Cook et al., 2021), but are nonetheless important to address in future research.

### Practice implications

The results of this review highlighted that, despite misconceptions around their desires, autistic adults do experience loneliness. Thus, it is important for clinical professionals to be vigilant in detecting the characteristics of loneliness in autistic adults (e.g. lack of relationships), which may or may not be attributed to their autism, to prevent associated negative outcomes, such as depression and suicide. The results of this review outline several factors that might be associated with loneliness in autistic people. It would be useful for clinicians to explore with their clients whether loneliness is due to autistic characteristics and/or environmental factors that could be overcome with appropriate accommodations. This review also helps to identify potential ways to support autistic adults who are lonely, such as through social groups (Bourdeau, 2020; Southby & Robinson, 2018).

## Conclusion

Research on loneliness in autistic adults is in its infancy. While there were limitations of the studies included within this review, it represents an important first step towards a more comprehensive understanding of loneliness in autistic adults. It highlights how loneliness and the desire for social connection are shared human experiences, regardless of whether a person is autistic or not. While the consequences of loneliness in autistic adults appear to be similar to those in the non-autistic population, it has not been established whether the mechanisms underpinning loneliness in autistic adults differ from non-autistic people. Further research on the topic is needed to better understand loneliness in autistic adults, focusing on the diversity of samples; the measures used to assess loneliness and the relationships examined in relation to loneliness. Underpinning all of this work should be the goal of making this research maximally beneficial to the lives of autistic adults (Pellicano et al., 2014).

## Supplemental Material

sj-docx-1-aut-10.1177_13623613221077721 – Supplemental material for Loneliness in autistic adults: A systematic review Click here for additional data file. Supplemental material, sj-docx-1-aut-10.1177_13623613221077721 for Loneliness in autistic adults: A systematic review by Kana Umagami, Anna Remington, Brynmor Lloyd-Evans, Jade Davies and Laura Crane in Autism

## Notes

1. Many members of the autistic and broader autism communities prefer identity-first language (i.e. autistic person), as they consider person-first language (i.e. person with autism) to be ableist and to perpetuate stigma (Bottema-Beutel et al., 2020; Gernsbacher, 2017; Kenny et al., 2016; Sinclair, 2013). We therefore use identity-first language throughout this review.

2. Self-identified autistic adults are those who identify as autistic but do not have a formal diagnosis. For example, they may be waiting to get a diagnosis, may not seek a diagnosis or may have difficulty accessing a diagnostic assessment (Lewis, 2016).

3. On the MMAT, the criterion on participants’ representativeness of the target population was included only in three study designs: quantitative non-randomized, quantitative descriptive and mixed methods studies.

4. A range of correlation coefficients were presented for the correlation between loneliness and social anxiety in Schiltz et al. (2020), due to two different scales of social anxiety being used in the study.

5. *Asperger United* is a quarterly newsletter published by National Autistic Society (a leading autism charity in the United Kingdom). It is written and edited by autistic people, with occasional contributions from professionals who work with autistic people.
