# Supplementary material for: Loneliness in autistic adults: A systematic review
Source: Autism. 2022 Mar 8;26(8):2117–35. doi: 10.1177/13623613221077721 (PMC9597154; doi:10.1177/13623613221077721)
Supplement: sj-docx-1-aut-10.1177_13623613221077721 – Supplemental material for Loneliness in autistic adults: A systematic review [file sj-docx-1-aut-10.1177_13623613221077721.docx]

Appendix A.

*Search strategy*

| **Database** | **Terms** | **Search within** | **Results** |
| --- | --- | --- | --- |
| ASSIA: Applied Social Sciences Index and Abstracts (ProQuest) | (autis* OR Asperger* OR Pervasive developmental disorder OR PDD OR ASD OR ASC) AND (lonel*OR social isolation) AND adult* | Abstract  (search was limited to abstracts because searching anywhere brought 2,838 results with too many irrelevant papers) | 29 |
|  | (autism AND loneliness AND adults) | Anywhere | 638 |
| BEI: British Education Index (EBSCO) | (autis* OR Asperger* OR Pervasive developmental disorder OR PDD OR ASD OR ASC) AND (lonel*OR social isolation) AND adult* | Not selected (all fields) | 18 |
|  | (autism AND loneliness AND adults) | Not selected (all fields) | 7 |
|  |  |  |  |
| ERIC (ProQuest) | (autis* OR Asperger* OR Pervasive developmental disorder OR PDD OR ASD OR ASC) AND (lonel*OR social isolation) AND adult* | Anywhere | 50 |
|  | (autism AND loneliness AND adults) | Anywhere | 12 |
|  |  |  |  |
| Medline (Ovid) | (autis* OR Asperger* OR Pervasive developmental disorder OR PDD OR ASD OR ASC) AND (lonel*OR social isolation) AND adult* | All fields | 97 |
|  | (autism AND loneliness AND adults) | All fields | 21 |
| ProQuest Dissertations and Thesis Global (ProQuest) | (autis* OR Asperger* OR Pervasive developmental disorder OR PDD OR ASD OR ASC) AND (lonel*OR social isolation) AND adult* | Abstract  (Search was limited to abstracts because searching anywhere brought 93,886 results with too many irrelevant papers. Search included both Master’s theses and Doctoral dissertations.) | 41 |
|  | (autism AND loneliness AND adults) | Abstract  (Search was limited to abstracts because searching anywhere brought 8,392 results with too many irrelevant papers. Search included both Master’s theses and Doctoral dissertations.) | 8 |
| PsycINFO | (autis* OR Asperger* OR Pervasive developmental disorder OR PDD OR ASD OR ASC) AND (lonel*OR social isolation) AND adult* | Abstracts  (Search was limited to abstracts because searching anywhere brought 4,235 results with too many irrelevant papers.) | 63 |
|  | (autism AND loneliness AND adults) | Abstracts  (Search was limited to abstracts because searching anywhere brought 2,180 results with too many irrelevant papers) | 24 |
| Scopus | (autis* OR Asperger* OR Pervasive developmental disorder OR PDD OR ASD OR ASC) AND (lonel*OR social isolation) AND adult* | Abstract  (Search was limited to abstracts because searching in all field brought 10,307 results with too many irrelevant papers) | 89 |
|  | (autism AND loneliness AND adults) | Abstract  (Search was limited to abstracts because searching in all field brought 3,300 results with too many irrelevant papers) | 24 |
| Web of Science Core Collection | (autis* OR Asperger* OR Pervasive developmental disorder OR PDD OR ASD OR ASC) AND (lonel*OR social isolation) AND adult* | All fields | 218 |
|  | (autism AND loneliness AND adults) | All fields | 117 |

Appendix B.

*List of the excluded studies at full-text assessment*

| **#** | **Study** | **Reasons for exclusion** |
| --- | --- | --- |
| 1 | Allely, 2015 | It was a review. |
| 2 | Bailey et al., 2020 | It did not report quantitative or qualitative data on loneliness. |
| 3 | Bal et al., 2021 | It did not report quantitative or qualitative data on loneliness. |
| 4 | Brooks & Paterson, 2011 | It did not report quantitative or qualitative data on loneliness. |
| 5 | Buonaguro & Bertelli, 2021 | Full text not available |
| 6 | Cage et al., 2018 | It did not report quantitative or qualitative data on loneliness. |
| 7 | Cai & Richdale, 2016 | It did not report quantitative or qualitative data on loneliness. |
| 8 | Cassidy et al., 2014 | It did not report quantitative or qualitative data on loneliness. |
| 9 | Causton-Theoharis et al., 2009 | It included the autobiographies that had a "focus on childhood experiences" (p. 86). |
| 10 | Churchard et al., 2019 | The participants did not have autism diagnosis. |
| 11 | Connor et al., 2020 | It did not report quantitative or qualitative data on loneliness. |
| 12 | Feldhaus et al., 2015 | The mean age of autistic participants was not reported though they included participants who were 15-27 years of age. The study referred to the participants as ‘youth’, ‘young people’ and ‘adolescents’. |
| 13 | Friedman et al., 2019 | It did not report quantitative or qualitative data on loneliness. |
| 14 | Furuhashi & Furuhashi, 2015 | Full text not available |
| 15 | Gelbar et al., 2014 | It was a review. |
| 16 | Happe & Charlton, 2012 | It was a review. |
| 17 | Howlin et al., 2004 | It was an editorial and did not constitute research. |
| 18 | Kapp et al., 2011 | It did not constitute research. |
| 19 | Kirby, 2016 | It did not report quantitative or qualitative data on loneliness. |
| 20 | Koegel et al., 2013 | It did not report quantitative or qualitative data on loneliness. |
| 21 | Laugeson et al., 2015 | It did not report quantitative or qualitative data on loneliness. |
| 22 | Lewis, 2016 | It did not report quantitative or qualitative data on loneliness. |
| 23 | Lucas & James, 2018 | It did not report quantitative or qualitative data on loneliness. |
| 24 | McConkey et al., 2021 | It did not report quantitative or qualitative data on loneliness. |
| 25 | McVey, 2016 | It is a thesis with the same study as MacVey et al. (2016). |
| 26 | Morrison et al., 2020 | It did not report quantitative or qualitative data on loneliness. |
| 27 | Simmons, 2020 | It did not constitute research. |
| 28 | Spain & Blainey, 2015 | It was a review. |
| 29 | Stice & Lavner, 2019 | Participants were not ‘autistic’ (not diagnosed or self-identified) and only had Broader Autism Phenotype. |
| 30 | Wendler, 2019 | Mean age of the participants were 15. |
| 31 | Ya-Ching & Ling-Yi, 2018 | It was a missed duplicate of Syu & Lin (2018). |

Appendix C.

*MMAT checklist table*

| Study | Category of study design | Screening questions and methodological quality criteria | Responses | | | | | | |
| --- | --- | --- | --- | --- | --- | --- | --- | --- | --- |
|  |  |  | Yes | No | | Can’t tell | | Comments | |
| Ashbaugh et al., 2017 | 3. Quantitative non-randomized | S1.^[[1]](#footnote-1)^ | ✓ |  | |  | |  | |
|  |  | S2. | ✓ |  | |  | |  | |
|  |  | 3.1. | ✓ |  | |  | | Clear descriptions of the target population and inclusion/exclusion criteria. (The sample does not appear to be the representation of the diverse autistic adults. ) | |
|  |  | 3.2. | ✓ |  | |  | | Interventions included engaging in social activities, social skill trainings, and peer mentoring by NT peer mentors. The numbers of such social engagements were self-reported every week during the intervention and the three-week follow-up. | |
|  |  | 3.3. | ✓ |  | |  | |  | |
|  |  | 3.4. |  | ✓ | |  | | It is hard to tell whether there were any other factors linked to the results without further qualitative exploration. | |
|  |  | 3.5. | ✓ |  | |  | |  | |
| Study | Category of study design | Screening questions and methodological quality criteria | Responses | | | | | | |
|  |  |  | Yes | No | | Can’t tell | | Comments | |
| Baldwin & Costley, 2016 | 5. Mixed Methods | S1. | ✓ |  | |  | |  | |
|  |  | S2. | ✓ |  | |  | |  | |
|  |  | 5.1. | ✓ |  | |  | |  | |
|  |  | 5.2. | ✓ |  | |  | |  | |
|  |  | 5.3. | ✓ |  | |  | |  | |
|  |  | 5.4. | ✓ |  | |  | | There is no divergence. | |
|  |  | 5.5. | ✓ |  | |  | | Clear descriptions of the target population and inclusion/exclusion criteria. (The sample does not appear to be the representation of the diverse autistic adults.) | |
| Study | Category of study design | Screening questions and methodological quality criteria | Responses | | | | | | |
|  |  |  | Yes | | No | | Can’t tell | | Comments |
| Bourdeau, 2020 (Doctoral thesis) | 3. Quantitative – non-randomized | S1. | ✓ | |  | |  | |  |
|  |  | S2. | ✓ | |  | |  | |  |
|  |  | 3.1. |  | | ✓ | |  | | The inclusion/exclusion criteria as well as the target population were not clear. (The sample does not appear to be the representation of the diverse autistic adults.) |
|  |  | 3.2. | ✓ | |  | |  | |  |
|  |  | 3.3. | ✓ | |  | |  | |  |
|  |  | 3.4. | ✓ | |  | |  | | No confounding expected |
|  |  | 3.5. | ✓ | |  | |  | |  |
| Study | Category of study design | Screening questions and methodological quality criteria | Responses | | | | | | |
|  |  |  | Yes | No | | Can’t tell | | Comments | |
| Brooks, 2014 (Doctoral thesis) | 4. Quantitative descriptive | S1. | ✓ |  | |  | |  | |
|  |  | S2. | ✓ |  | |  | |  | |
|  |  | 4.1. | ✓ |  | |  | |  | |
|  |  | 4.2. | ✓ |  | |  | | Clear descriptions of the target population and inclusion/exclusion criteria. (The sample does not appear to be the representation of the diverse autistic adults.) | |
|  |  | 4.3. | ✓ |  | |  | |  | |
|  |  | 4.4. |  |  | | ✓ | | Response rate was not reported. | |
|  |  | 4.5. | ✓ |  | |  | | A clear explanation on analysis was not found, but the statistics were presented in the texts and tables. | |
| Study | Category of study design | Screening questions and methodological quality criteria | Responses | | | | | | |
|  |  |  | Yes | No | | Can’t tell | | Comments | |
| Caruana et al., 2021 | 4. Quantitative descriptive | S1. | ✓ |  | |  | |  | |
|  |  | S2. | ✓ |  | |  | |  | |
|  |  | 4.1. | ✓ |  | |  | |  | |
|  |  | 4.2. | ✓ |  | |  | | Clear descriptions of the target population and inclusion/exclusion criteria. (The sample does not appear to be the representation of the diverse autistic adults.) | |
|  |  | 4.3. | ✓ |  | |  | |  | |
|  |  | 4.4. | ✓ |  | |  | |  | |
|  |  | 4.5. | ✓ |  | |  | |  | |
| Study | Category of study design | Screening questions and methodological quality criteria | Responses | | | | | | |
|  |  |  | Yes | No | | Can’t tell | | Comments | |
| Chen et al., 2016 | 4. Quantitative descriptive | S1. | ✓ |  | |  | |  | |
|  |  | S2. | ✓ |  | |  | |  | |
|  |  | 4.1. | ✓ |  | |  | |  | |
|  |  | 4.2. | ✓ |  | |  | | Clear descriptions of the target population and inclusion/exclusion criteria. (The sample does not appear to be the representation of the diverse autistic adults.) | |
|  |  | 4.3. | ✓ |  | |  | |  | |
|  |  | 4.4. | ✓ |  | |  | |  | |
|  |  | 4.5. | ✓ |  | |  | |  | |
| Study | Category of study design | Screening questions and methodological quality criteria | Responses | | | | | | |
|  |  |  | Yes | No | | Can’t tell | | Comments | |
| Ee et al. 2019 | 5. Mixed methods | S1. | ✓ |  | |  | |  | |
|  |  | S2. | ✓ |  | |  | |  | |
|  |  | 5.1. | ✓ |  | |  | | The rationale for using a mixed methods design was clear in the aim of this study. | |
|  |  | 5.2. | ✓ |  | |  | | Both quantitative and qualitative data contributed to the whole picture of the results from this study. | |
|  |  | 5.3. | ✓ |  | |  | | Social aspect relating to the experiences of loneliness were explored with both quantitative and qualitative data collection. | |
|  |  | 5.4. | ✓ |  | |  | | There was no divergence between quantitative and qualitative results. | |
|  |  | 5.5. | ✓ |  | |  | | The study was overall a high quality according to the criteria for both methods. (The sample does not appear to be the representation of the diverse autistic adults.) | |
| Study | Category of study design | Screening questions and methodological quality criteria | Responses | | | | | | |
|  |  |  | Yes | | No | | Can’t tell | | Comments |
| Elmose 2020 | 1. Qualitative | S1. | ✓ | |  | |  | |  |
|  |  | S2. | ✓ | |  | |  | |  |
|  |  | 1.1. | ✓ | |  | |  | | Among the 25 participants, 24 of them involved in focus group interviews and four autistic adults also involved in individual interviews. |
|  |  | 1.2. | ✓ | |  | |  | | Clear justification was made to explain why the study used both focus group interviews and individual interviews. |
|  |  | 1.3. | ✓ | |  | |  | | Phenomenological perspective using thematic analysis was rightfully used. |
|  |  | 1.4. | ✓ | |  | |  | |  |
|  |  | 1.5. | ✓ | |  | |  | |  |
| Study | Category of study design | Screening questions and methodological quality criteria | Responses | | | | | | |
|  |  |  | Yes | No | | Can’t tell | | Comments | |
| Gantman et al., 2012 | 2. Quantitative randomized controlled trials | S1. | ✓ |  | |  | |  | |
|  |  | S2. | ✓ |  | |  | |  | |
|  |  | 2.1. | ✓ |  | |  | | They flipped a coin for randomization. | |
|  |  | 2.2 | ✓ |  | |  | |  | |
|  |  | 2.3. | ✓ |  | |  | |  | |
|  |  | 2.4 | ✓ |  | |  | |  | |
|  |  | 2.5. | ✓ |  | |  | | All of their participants (N=17) continued the assigned intervention. | |
| Study | Category of study design | Screening questions and methodological quality criteria | Responses | | | | | | |
|  |  |  | Yes | | No | | Can’t tell | | Comments |
| Han et al. 2019 | 4. Quantitative descriptive studies | S1. | ✓ | |  | |  | |  |
|  |  | S2. | ✓ | |  | |  | |  |
|  |  | 4.1. | ✓ | |  | |  | | They made sure that the participants were appropriately assigned to the three groups in the study: ASD, currently depressed and control groups. (The sample does not appear to be the representation of the diverse autistic adults.) |
|  |  | 4.2. | ✓ | |  | |  | | Clear descriptions of the target population and inclusion/exclusion criteria. |
|  |  | 4.3 | ✓ | |  | |  | |  |
|  |  | 4.4. | ✓ | |  | |  | |  |
|  |  | 4.5. | ✓ | |  | |  | | They provided reasons for each analysis. |
| Study | Category of study design | Screening questions and methodological quality criteria | Responses | | | | | | |
|  |  |  | Yes | No | | Can’t tell | | Comments | |
| Hedley et al., 2018a | 4. Quantitative descriptive | S1. | ✓ |  | |  | |  | |
|  |  | S2. | ✓ |  | |  | |  | |
|  |  | 4.1. |  | ✓ | |  | | A clear justification of the sample frame used is not provided | |
|  |  | 4.2. | ✓ |  | |  | | Clear description of inclusion criteria despite no clear descriptions of the target population. (The sample does not appear to be the representation of the diverse autistic adults.) | |
|  |  | 4.3. | ✓ |  | |  | |  | |
|  |  | 4.4. | ✓ |  | |  | |  | |
|  |  | 4.5. | ✓ |  | |  | |  | |
| Study | Category of study design | Screening questions and methodological quality criteria | Responses | | | | | | |
|  |  |  | Yes | No | | Can’t tell | | Comments | |
| Hedley et al., 2018b | 4.Quantitative descriptive | S1. | ✓ |  | |  | |  | |
|  |  | S2. | ✓ |  | |  | |  | |
|  |  | 4.1. | ✓ |  | |  | |  | |
|  |  | 4.2. | ✓ |  | |  | | Clear inclusion criteria for participant despite no clear descriptions of the target population. (The sample does not appear to be the representation of the diverse autistic adults.) | |
|  |  | 4.3. | ✓ |  | |  | |  | |
|  |  | 4.4. | ✓ |  | |  | |  | |
|  |  | 4.5. | ✓ |  | |  | |  | |
| Study | Category of study design | Screening questions and methodological quality criteria | Responses | | | | | | |
|  |  |  | Yes | No | | Can’t tell | | Comments | |
| Hickey et al., 2018 | 1.Qualitative | S1. | ✓ |  | |  | |  | |
|  |  | S2. | ✓ |  | |  | |  | |
|  |  | 1.1. | ✓ |  | |  | | Appropriate to investigate the experiences of older autistic adults. | |
|  |  | 1.2. | ✓ |  | |  | | Semi-structured interviews were used. | |
|  |  | 1.3. | ✓ |  | |  | | Thematic analysis was used. | |
|  |  | 1.4. | ✓ |  | |  | | They provided quotes from the participants to justify each theme. | |
|  |  | 1.5. | ✓ |  | |  | |  | |
| Study | Category of study design | Screening questions and methodological quality criteria | Responses | | | | | | |
|  |  |  | Yes | No | | Can’t tell | | Comments | |
| Hillier et al., 2018 | 5. Mixed methods | S1. | ✓ |  | |  | |  | |
|  |  | S2. | ✓ |  | |  | |  | |
|  |  | 5.1. |  |  | | ✓ | | While the mixed method appears to be appropriate in this study, a clear rationale is not found in the text. | |
|  |  | 5.2. |  | ✓ | |  | | Qualitative and quantitative results weren’t effectively integrated. | |
|  |  | 5.3. |  | ✓ | |  | | It is not clear how the authors attempted to draw the results across qualitative and quantitative components. | |
|  |  | 5.4. |  |  | | ✓ | |  | |
|  |  | 5.5. | ✓ |  | |  | | The study was overall a high quality according to the criteria for both methods. (The sample does not appear to be the representation of the diverse autistic adults.) | |
| Study | Category of study design | Screening questions and methodological quality criteria | Responses | | | | | | |
|  |  |  | Yes | No | | Can’t tell | | Comments | |
| Hull et al., 2017 | 1.Qualitative | S1. | ✓ |  | |  | |  | |
|  |  | S2. | ✓ |  | |  | |  | |
|  |  | 1.1. | ✓ |  | |  | |  | |
|  |  | 1.2. | ✓ |  | |  | |  | |
|  |  | 1.3. | ✓ |  | |  | | Findings are derived from the data by thematic analysis using inductive approach. | |
|  |  | 1.4. | ✓ |  | |  | | They provided quotes from the participants to justify each theme. | |
|  |  | 1.5. | ✓ |  | |  | |  | |
| Study | Category of study design | Screening questions and methodological quality criteria | Responses | | | | | | |
|  |  |  | Yes | No | | Can’t tell | | Comments | |
| Hwang et al., 2017 | 1. Qualitative | S1. | ✓ |  | |  | |  | |
|  |  | S2. | ✓ |  | |  | |  | |
|  |  | 1.1. | ✓ |  | |  | |  | |
|  |  | 1.2. | ✓ |  | |  | |  | |
|  |  | 1.3. | ✓ |  | |  | |  | |
|  |  | 1.4. | ✓ |  | |  | |  | |
|  |  | 1.5. | ✓ |  | |  | |  | |
| Study | Category of study design | Screening questions and methodological quality criteria | Responses | | | | | | |
|  |  |  | Yes | No | | Can’t tell | | Comments | |
| Jackson et al., 2018 | 4.Quantitative descriptive | S1. | ✓ |  | |  | |  | |
|  |  | S2. | ✓ |  | |  | |  | |
|  |  | 4.1. | ✓ |  | |  | |  | |
|  |  | 4.2. |  |  | | ✓ | | The inclusion/exclusion criteria were not clear, but the target population was clear. (The sample does not appear to be the representation of the diverse autistic adults.) | |
|  |  | 4.3. | ✓ |  | |  | |  | |
|  |  | 4.4. |  |  | | ✓ | | The information needed to judge this criterion is not reported in the study (i.e. response rate is not reported). | |
|  |  | 4.5. | ✓ |  | |  | |  | |
| Study | Category of study design | Screening questions and methodological quality criteria | Responses | | | | | | |
|  |  |  | Yes | No | | Can’t tell | | Comments | |
| Jantz, 2011 | 5. Mixed methods | S1. | ✓ |  | |  | |  | |
|  |  | S2. | ✓ |  | |  | |  | |
|  |  | 5.1. |  | ✓ | |  | | The reasons for conducting a mixed methods study was not clearly explained. | |
|  |  | 5.2. | ✓ |  | |  | |  | |
|  |  | 5.3. | ✓ |  | |  | | The findings were interpreted based on both quantitative and qualitative components. | |
|  |  | 5.4. | ✓ |  | |  | | There were no divergences and inconsistencies between quantitative and qualitative result. | |
|  |  | 5.5. | ✓ |  | |  | | The inclusion/exclusion criteria were not clear but the target population was clear. Overall, the study was a high quality according to the criteria for both methods. (The sample does not appear to be the representation of the diverse autistic adults.) | |
| Study | Category of study design | Screening questions and methodological quality criteria | Responses | | | | | | |
|  |  |  | Yes | | No | | Can’t tell | | Comments |
| Levinson, 2020 (Doctoral thesis) | 4. Quantitative descriptive | S1. | ✓ | |  | |  | |  |
|  |  | S2. | ✓ | |  | |  | |  |
|  |  | 4.1. |  | |  | | ✓ | | It was not clear where the study recruited the participants. |
|  |  | 4.2. |  | | ✓ | |  | | No clear inclusion/exclusion criteria and no clear descriptions of the target population. (The sample does not appear to be the representation of the diverse autistic adults.) |
|  |  | 4.3. | ✓ | |  | |  | | Validated and reliable measures were used and the measurements were justified. |
|  |  | 4.4. | ✓ | |  | |  | |  |
|  |  | 4.5. | ✓ | |  | |  | |  |
| Study | Category of study design | Screening questions and methodological quality criteria | Responses | | | | | | |
|  |  |  | Yes | No | | Can’t tell | | Comments | |
| Lin & Huang, 2017 | 4. Quantitative descriptive | S1. | ✓ |  | |  | |  | |
|  |  | S2. | ✓ |  | |  | |  | |
|  |  | 4.1. | ✓ |  | |  | |  | |
|  |  | 4.2. | ✓ |  | |  | | Clear descriptions of the target population and inclusion/exclusion criteria. (The sample does not appear to be the representation of the diverse autistic adults.) | |
|  |  | 4.3. | ✓ |  | |  | |  | |
|  |  | 4.4. | ✓ |  | |  | |  | |
|  |  | 4.5. | ✓ |  | |  | | Clear justification for analyses was provided. | |
| Study | Category of study design | Screening questions and methodological quality criteria | Responses | | | | | | |
|  |  |  | Yes | No | | Can’t tell | | Comments | |
| Mazurek, 2013 | 5. Mixed methods | S1. | ✓ |  | |  | |  | |
|  |  | S2. | ✓ |  | |  | |  | |
|  |  | 5.1. | ✓ |  | |  | |  | |
|  |  | 5.2. | ✓ |  | |  | |  | |
|  |  | 5.3. | ✓ |  | |  | |  | |
|  |  | 5.4. | ✓ |  | |  | | There were no divergences and inconsistencies between quantitative and qualitative results. | |
|  |  | 5.5. | ✓ |  | |  | | The inclusion/exclusion criteria were no clear, but overall the study was a high quality according to the criteria for both methods. | |
| Study | Category of study design | Screening questions and methodological quality criteria | Responses | | | | | | |
|  |  |  | Yes | No | | Can’t tell | | Comments | |
| Mazurek, 2014 | 4. Quantitative descriptive | S1. | ✓ |  | |  | |  | |
|  |  | S2. | ✓ |  | |  | |  | |
|  |  | 4.1. | ✓ |  | |  | |  | |
|  |  | 4.2. | ✓ |  | |  | | Clear descriptions of the target population and inclusion/exclusion criteria. (The sample does not appear to be the representation of the diverse autistic adults.) | |
|  |  | 4.3. | ✓ |  | |  | |  | |
|  |  | 4.4. |  |  | | ✓ | | Response rate was not reported, and indicators of low nonresponse bias were also not presented. | |
|  |  | 4.5. | ✓ |  | |  | |  | |
| Study | Category of study design | Screening questions and methodological quality criteria | Responses | | | | | | |
|  |  |  | Yes | No | | Can’t tell | | Comments | |
| McVey et al., 2016 | 2. Quantitative randomised controlled trial | S1. | ✓ |  | |  | |  | |
|  |  | S2. | ✓ |  | |  | |  | |
|  |  | 2.1. |  | ✓ | |  | | It did not provide a description on how the randomization schedule was generated. | |
|  |  | 2.2. | ✓ |  | |  | |  | |
|  |  | 2.3. | ✓ |  | |  | |  | |
|  |  | 2.4. | ✓ |  | |  | |  | |
|  |  | 2.5. | ✓ |  | |  | |  | |
| Study | Category of study design | Screening questions and methodological quality criteria | Responses | | | | | | |
|  |  |  | Yes | No | | Can’t tell | | Comments | |
| Merkler, 2007 (Doctoral thesis) | 4. Quantitative descriptive | S1. | ✓ |  | |  | |  | |
|  |  | S2. | ✓ |  | |  | |  | |
|  |  | 4.1. | ✓ |  | |  | |  | |
|  |  | 4.2. |  | ✓ | |  | | Inclusion and exclusion criteria were not clearly stated. | |
|  |  | 4.3. | ✓ |  | |  | |  | |
|  |  | 4.4. | ✓ |  | |  | |  | |
|  |  | 4.5. | ✓ |  | |  | |  | |
| Study | Category of study design | Screening questions and methodological quality criteria | Responses | | | | | | |
|  |  |  | Yes | No | | Can’t tell | | Comments | |
| Milton & Sims, 2016 | 1.Qualitative | S1. | ✓ |  | |  | |  | |
|  |  | S2. | ✓ |  | |  | |  | |
|  |  | 1.1. | ✓ |  | |  | | The study explored the narratives of autistic adults within the autism-related magazine. | |
|  |  | 1.2. | ✓ |  | |  | |  | |
|  |  | 1.3. | ✓ |  | |  | |  | |
|  |  | 1.4. | ✓ |  | |  | |  | |
|  |  | 1.5. | ✓ |  | |  | |  | |
| Study | Category of study design | Screening questions and methodological quality criteria | Responses | | | | | | |
|  |  |  | Yes | No | | Can’t tell | | Comments | |
| Orsmond et al., 2013 | 4. Quantitative descriptive | S1. | ✓ |  | |  | |  | |
|  |  | S2. | ✓ |  | |  | |  | |
|  |  | 4.1. | ✓ |  | |  | |  | |
|  |  | 4.2. |  |  | | ✓ | | The participants were recruited from a longitudinal study, the inclusion/exclusion criteria were not clear. | |
|  |  | 4.3. | ✓ |  | |  | | Measures were not what were validated; however, the variables were clearly defined within their own quantitative questionnaires. | |
|  |  | 4.4. | ✓ |  | |  | | The data were from a national study. | |
|  |  | 4.5. | ✓ |  | |  | |  | |
| Study | Category of study design | Screening questions and methodological quality criteria | Responses | | | | | | |
|  |  |  | Yes | No | | Can’t tell | | Comments | |
| Russell, 2020 | 4. Quantitative descriptive | S1. | ✓ |  | |  | |  | |
|  |  | S2. | ✓ |  | |  | |  | |
|  |  | 4.1. | ✓ |  | |  | |  | |
|  |  | 4.2. | ✓ |  | |  | | Clear descriptions of the target population and inclusion/exclusion criteria. (The sample does not appear to be the representation of the diverse autistic adults.) | |
|  |  | 4.3. | ✓ |  | |  | |  | |
|  |  | 4.4. | ✓ |  | |  | |  | |
|  |  | 4.5. | ✓ |  | |  | |  | |
| Study | Category of study design | Screening questions and methodological quality criteria | Responses | | | | | | |
|  |  |  | Yes | No | | Can’t tell | | Comments | |
| Schiltz et al., 2020 | 4. Quantitative descriptive | S1. | ✓ |  | |  | |  | |
|  |  | S2. | ✓ |  | |  | |  | |
|  |  | 4.1. | ✓ |  | |  | | The source of sample and sampling procedure appear to be adequate. | |
|  |  | 4.2. | ✓ |  | |  | | Clear descriptions of the target population and inclusion criteria. | |
|  |  | 4.3 | ✓ |  | |  | | They used appropriate measures that were validated and reliable. | |
|  |  | 4.4. | ✓ |  | |  | |  | |
|  |  | 4.5 | ✓ |  | |  | |  | |
| Study | Category of study design | Screening questions and methodological quality criteria | Responses | | | | | | |
|  |  |  | Yes | No | | Can’t tell | | Comments | |
| Smith & Sharp, 2013 | 1.Qualitative | S1. | ✓ |  | |  | |  | |
|  |  | S2. | ✓ |  | |  | |  | |
|  |  | 1.1. | ✓ |  | |  | | The study explored the unique sensory experiences of autistic adults using the Ground Theory. | |
|  |  | 1.2. | ✓ |  | |  | |  | |
|  |  | 1.3. | ✓ |  | |  | |  | |
|  |  | 1.4. | ✓ |  | |  | |  | |
|  |  | 1.5. | ✓ |  | |  | |  | |
| Study | Category of study design | Screening questions and methodological quality criteria | Responses | | | | | | |
|  |  |  | Yes | No | | Can’t tell | | Comments | |
| Southby & Robinson, 2018 | 1.Qualitative | S1. | ✓ |  | |  | |  | |
|  |  | S2. | ✓ |  | |  | |  | |
|  |  | 1.1. | ✓ |  | |  | | The rationale was clearly stated. | |
|  |  | 1.2. | ✓ |  | |  | | Semi-structured interviewed were conducted to collect the data. | |
|  |  | 1.3. | ✓ |  | |  | |  | |
|  |  | 1.4. | ✓ |  | |  | |  | |
|  |  | 1.5. | ✓ |  | |  | |  | |
| Study | Category of study design | Screening questions and methodological quality criteria | Responses | | | | | | |
|  |  |  | Yes | No | | Can’t tell | | Comments | |
| Sundberg, 2018 | 4. Quantitative descriptive | S1. | ✓ |  | |  | |  | |
|  |  | S2. | ✓ |  | |  | |  | |
|  |  | 4.1. | ✓ |  | |  | |  | |
|  |  | 4.2. | ✓ |  | |  | | Clear descriptions of the target population and inclusion/exclusion criteria. (The sample does not appear to be the representation of the diverse autistic adults.) | |
|  |  | 4.3 | ✓ |  | |  | |  | |
|  |  | 4.4. | ✓ |  | |  | |  | |
|  |  | 4.5. | ✓ |  | |  | |  | |
| Study | Category of study design | Screening questions and methodological quality criteria | Responses | | | | | | |
|  |  |  | Yes | No | | Can’t tell | | Comments | |
| Syu & Lin, 2018 | 4. Quantitative descriptive | S1. | ✓ |  | |  | |  | |
|  |  | S2. | ✓ |  | |  | |  | |
|  |  | 4.1. | ✓ |  | |  | |  | |
|  |  | 4.2. | ✓ |  | |  | | Clear descriptions of the target population and inclusion/exclusion criteria. (The sample does not appear to be the representation of the diverse autistic adults.) | |
|  |  | 4.3. | ✓ |  | |  | |  | |
|  |  | 4.4. |  |  | | ✓ | | Response rate was not reported, and indicators of low nonresponse bias were also not presented. | |
|  |  | 4.5. | ✓ |  | |  | |  | |
| Study | Category of study design | Screening questions and methodological quality criteria | Responses | | | | | | |
|  |  |  | Yes | No | | Can’t tell | | Comments | |
| Van der Aa et al., 2016 | 5. Mixed methods | S1. | ✓ |  | |  | |  | |
|  |  | S2. | ✓ |  | |  | |  | |
|  |  | 5.1. | ✓ |  | |  | | The reason for using this particular method was not clearly explained. | |
|  |  | 5.2. | ✓ |  | |  | |  | |
|  |  | 5.3. | ✓ |  | |  | |  | |
|  |  | 5.4. | ✓ |  | |  | | No divergences and inconsistencies between quantitative and qualitative results were found. | |
|  |  | 5.5 | ✓ |  | |  | | Overall, the study was a high quality according to the criteria for both methods. | |
| Study | Category of study design | Screening questions and methodological quality criteria | Responses | | | | | | |
|  |  |  | Yes | No | | Can’t tell | | Comments | |
| Van Hees et al., 2015 | 1.Qualitative | S1. | ✓ |  | |  | |  | |
|  |  | S2. | ✓ |  | |  | |  | |
|  |  | 1.1. | ✓ |  | |  | | The study explored the higher education experiences of autistic students. | |
|  |  | 1.2. | ✓ |  | |  | | Semi-structured interviews were used. | |
|  |  | 1.3. | ✓ |  | |  | |  | |
|  |  | 1.4. | ✓ |  | |  | |  | |
|  |  | 1.5. | ✓ |  | |  | |  | |

Appendix D.

*Characteristics of 34 included studies*

| **Study** | **Origin** | **Study Design** | **N (Male/Female/other)** | **Age**  **(Range, M, SD)** | **Study Description** | **Key Outcomes** |
| --- | --- | --- | --- | --- | --- | --- |
| Ashbaugh et al., 2017 | USA | Quantitative non-randomized (multiple case studies) | 3 (2/1/NR) | 19-24, NR, NR | Social intervention program with weekly meetings and peer mentoring. | Increased social integration, academic performance and satisfaction with university experience. |
| Baldwin & Costley, 2016 | Australia | Mixed Methods (nation-wide survey) | 82 (NA, 82, NA) | 18-64, 32.7, 12.3 | Self-report on health, work, education, social and community activities. | Poor mental health, unmet needs of support in education and employment, and social isolation were highlighted. |
| Bourdeau, 2020 (Doctoral thesis) | USA | Quantitative – non-randomized (quasi-experimental study) | Adults with ASD = 38, their caregivers = 38 | Adults with ASD = 18-40, NR, NR | Quasi-experimental study to measure the impacts of social group participation on autism symptomology, social skills, and loneliness | Three-month engagement in social groups resulted in a significant decrease in the perceptions of ASD symptomology and social skills in both autistic adults and their caregivers, and a significant decrease in loneliness for autistic adults as a group. |
| Brooks, 2014 (Doctoral thesis) | USA | Quantitative descriptive (standardized questionnaires) | HFASD = 56 (28/28/NR), parent/caregiver = 56 (NR/NR/NR), typically-developing (TD) = 56 (28/28/NR) | HFASD = 18-40, 26.3, 6, TD = NR, 26.4, 4.6 | Dissertation investigating gender differences in social skills, peer relationships, and emotional correlates. | Higher level of quality in friendship is associated with lower level of loneliness in both HFASD and TD groups. |
| Caruana et al., 2021 | Australia, UK (Study 1 was conducted in both countries, Study 2 was with a smaller portion of participants who previously took part in the UK-based survey in Study 1) | Quantitative descriptive (online survey) | Study 1 = full sample: 870 (261, 592, 15, 2 preferred not to say), no diagnosis: 484(111, 372, 1), autistic: 281(122, 147, 10, 2 preferred not to say)  Study 2 = 89 (29, 51, 5, 4 preferred not to say) | Study 1 = full sample: 16-75+, no diagnosis: 16-75+, autistic: 16-somewhere between 65-74, Study 2 = 16-74 | Research based on two quantitative studies which explored the relationships between 1) autistic traits and anthropomorphism in the general population, and 2) loneliness and anthropomorphism in autistic adults. | More autistic traits, increased tendency to anthropomorphise non-human entities in the general population. More tendencies to anthropomorphism were associated with increased loneliness in autistic adults. |
| Chen et al., 2016 | Australia, Taiwan | Quantitative descriptive (experience sampling methodology (ESM)) | 30 (16/14/NR) | 16-45, 24.8 (Australian participants: n=14), 9.0 (Australian participants: n=14), 27.8 (Taiwanese participants: n=16), 6.3 (Taiwanese participants: n=16) | ESM surveys of everday activity of "high-functioning" autistic individuals for 7 days, 7 times/day. | Highlighted the importance of considering in-the-moment experience in autism research. Level of loneliness did not depend on the types of activites. |
| Ee et al., 2019 | Australia | Mixed methods Self-report (questionnaire and open-ended questionnaire) | Autistic = 220 (86/124/10), nonautistic = 146 (29/117/NR) | Autistic = 25-80, 41.9,  12.24, nonautistic = 25-79, 43.7, 13.49 | Mixed method study of associated factors of loneliness and experiences of loneliness among autistic adults, collecting data from a longitudinal study. | Autistic adults were significantly lonelier than nonautistic adults. Social skills and dissatisfaction with social support were related to loneliness in both autistic and nonautistic adults. Thematic analysis showed variable experiences and perceptions of socialization. |
| Elmose, 2020 | Denmark | Qualitative (focus groups and individual interviews) | 25 (18/7/NR) | 18-71, NR, NR | Qualitative study of the concept of loneliness among autistic adults. | Four themes were identified using a phenomenological thematic analysis: experience of loneliness, being autistic, discrepancies in social relationships, and ease of interaction. |
| Gantman et al., 2012 | USA | Quantitative randomized controlled trials (randomized control trial) | 17 (12/5/NR) | 18-23, 20.4, 1.62 | Social intervention Program using *The UCLA PEERS for Young Adults Program*. | PEERS social skill training improved caregiver-reported social skills of autistic young adults and it lessened social and emotional loneliness measured by SELSA. |
| Han et al., 2019 | USA | Quantitative descriptive studies (self-report questionnaire) | Typically developing (TD) control = 28 (14/14/0), ASD = 49 (31/18/0), TD currently depressed = 30 (12/18/0) | TD control = NR, 25.32, 5.28, ASD = NR, 23.98 26.23, = TD currently depressed = NR, 26.23 4.67 | Quantitative study of the relationships between self-reported pleasure in social and non-social rewards, severity of autism symptoms, loneliness and depressive symptoms in three groups: autistic adults, currently depressed adults and control group. | The less pleasure adults experienced, the higher loneliness they reported whether or not they were autistic. Autistic adults experienced high level of loneliness even when they reported high capacity for pleasure. Loneliness was the most reliable predictor of depression, compared to the capacity for pleasure and autism symptoms. |
| Hedley et al., 2018a | Australia | Quantitative descriptive (online survey) | 71 (63/8/NR) | 17-56, 26.14, 8.20 | Online survey of loneliness, depression, and thoughts of self-harm. | Loneliness is a risk factor for  depression and thoughts of self-  harm. Depression is not the indicator for  loneliness or thoughts of self-harm. |
| Hedley et al., 2018b | Australia | Quantitative descriptive (self-report questionnaire) | 185 (93/92/NR) | 14-80, 37.11, 15.41 | Self-report of loneliness, social support and suicidal ideation and depression. | For depression and suicidal ideation, loneliness is a risk factor and social support is a protective factor. |
| Hickey et al., 2018 | UK | Qualitative (semi-structured interview) | 13 (10/3/NR) | 51-71, NR, NR | Semi-structured interviews and thematic analysis of experiences of autistic adults in their late adulthood (pre- and post- diagnosis). | Highlighted difference in pre-diagnosis, life-review in post-diagnosis, and longing for connection throughout adulthood. |
| Hillier et al., 2018 | UK | Mixed methods (self-report questionnaires and focus groups) | 52 (51/1/NR) | 18-28, 20.9, NR (all university students) | Self-report of self-esteem, loneliness and mental health and focus group on the 7-weeks social intervention program. | The social intervention program  reduced loneliness  and general anxiety and  increased self-esteem.  However, it did not have impacts on social anxiety, academic distress or depression. |
| Hull et al., 2017 | UK | Qualitative (online survey) | 92 (55/30/7) | Female = 18-68, 40.71, 14.14, Male = 22-79, 48.03, 16.62, Others = 27-69, 40.71, 14.29 | Online survey of motivation, techniques and consequences of camouflaging. | Camouflaging is motivated by the desire to fit in and to have connections with others, presented as masking or compensation, and results in exhaustion, stress, anxiety followed by the need to be alone and identity crisis. |
| Hwang et al., 2017 | Australia | Qualitative (semi-structured interviews) | Autistic adults = 15 (11/4/0), Carers = 9 (1/8/0) | Autistic adults = NR/50.3/17.11, Carers = NR/63.6/8.76 | Semi-structured interviews with autistic adults and carers of autistic adults to explore the meaning of “age well” from their first point of views. | Thematic analysis identified eight themes: myself, being autistic, others, lifestyle and living well, being supported, relating to others, life environment, and societal attitudes and acceptance. |
| Jackson et al., 2018 | USA | Quantitative descriptive (online survey) | 56 (26/26/4) | 18-57, 22.98, 6.01 | Online survey of the experiences of autistic students in post-secondary education, specifically on the academic, social and mental health aspects. | Autistic students showed high  level of academic comfort while  they showed a high level of  isolation, loneliness, anxiety and depression. About three-quarters of them expressed their lifetime suicidal behaviours. |
| Jantz, 2011 | USA | Mixed methods (interviews and self-report questionnaire) | 35 (24/11/NR) | 24-77, NR, NR | Mixed method study on loneliness and the perception of support groups. | The participants  showed a higher level of loneliness  compared to their counterparts,  and they perceived support  groups as helpful due to  social skills and  support, information and advice,  and structure. |
| Levinson, 2020 (Doctoral thesis) | USA | Quantitative descriptive (self-report questionnaire) | ASD = 123 (28/59/32/gender not reported n= 4), neurotypical = 115 (25/72/15 gender not reported n= 3) | ASD = NR, 23.13 (3.58), neurotypical = NR, 22.92 (3.37) | Quantitative study on the relationships among distress, emotion regulation and self-harm among autistic and neurotypical young adults. | Autistic and neurotypical young adults were similar in how they engaged in self-injurious behaviours (SIBs) and how they affected them. While lack of communication skills, anxiety and depression were associated with SIBs, lack of social skills, loneliness, emotional dysregulation and restricted and repetitive behaviours were not associated with SIBs. |
| Lin & Huang, 2019 | Taiwan | Quantitative descriptive (interviews) | ASD (autism spectrum disorder) = 66 (43/23/NR), neuro-typical= 85 (52/33/NR) | ASD = 20-38, 27.8, 5.2, neurotypical = 20-38, 27.8, 4.3 | Comparison of QoL and its association with anxiety, loneliness and sensory processing between ASD and neuro-typical groups. | Adults with ASD show lower quality  of life, higher anxiety, higher  loneliness and more difficulty with  sensory processing than  neuro-typical adults. |
| Mazurek, 2013 | USA | Mixed methods (self-report questionnaire) | 108 (52.8%/47.2%/NR) | 18-62, 32.4, 12.5 | Self-report of social media use, friendships and loneliness. | 79.6% of the participants used  SNS and the most popular reason for the SNS use was for social conneciton. Those who used SNS were more liekly to report having close frineds. However, decreased loneliness is associated with the friendship quality and quantity offline and not online. |
| Mazurek, 2014 | USA | Quantitative descriptive (online self-report questionnaire) | 108 (52.8%/47.2%/NR) | 18-62, 32.4, 12.5 | Self-report of loneliness, friendship and well-being (self-esteem, depression, life-satisfaction). | Loneliness is positively  correlated with depression and  anxiety, and it is negatively  correlated with life-satisfaction  and self-esteem. Friendships and autistic characteristics are also highly correlated with loneliness. |
| McVey et al., 2016 | USA | Quantitative randomised controlled trial | Experimental = 24 (18/6/0), Waitlist control = 23 (20/3/0) | Experimental = NR/20.92/3.31, Waitlist control = NR/19.52/1.70 | Replication and extension of the Gantman et al. (2012) by also examining the effects of PEERS on social anxiety in autistic young adults. | Improvements due to the PEERS were seen in social responsiveness, PEERS knowledge, empathy, and social anxiety. |
| Merkler, 2007 (Doctoral thesis) | USA | Quantitative descriptive (self-report questionnaire) | high functioning autism = 37 (30/7/NR), = typically developing = 82 (26/56/NR) | 18-52, 29.65, 10.19 | Self-report of social relationships, loneliness and mental health. | More social isolation was seen in the adults “with high-functioning autism” than their peers, and isolation was associated with distress in the population. |
| Milton & Sims, 2016 | UK | Qualitative (thematic analysis of issues of the magazine, *Asperger United* (AU)) | NA | NA | Thematic analysis of issues of the magazine, *Asperger United* (AU). | Highlighted the importance of limiting social isolation of autistic adults. |
| Orsmond et al., 2013 | USA | Quantitative descriptive (telephone interview) | 620 (Wave 5 of the data from the National Longitudinal Transition Study 2) | 21-25, NR, NR | Telephone interviews with young adults “with ASD” or their parents/guardians on social participation of young adults “with ASD” in the past 12 months. | Compared to the other  disability groups (intellectual disability, emotional disturbance, learning disabilities), young  adults “with ASD” are likely to never see  friends, never get calls from  friends, never be invited to  activities and be socially  isolated. |
| Russell, 2020  (Doctoral thesis) | USA | Quantitative descriptive (self-report questionnaire) | Autistic group = 22 (6 female), Insomnia group = 23 (11 female), ‘NT’ (typically developing) group = 21 (13 female) | Autistic group = NR, 25.26, 3.97, Insomnia group = NR, 24.95, 4,64, Neurotypical group = NR, 23.55, 4.88, | Quantitative study of the association of sleep quality and loneliness with perceived physical and mental health. | Lower sleep quality and higher levels of loneliness were positively associated with physical and mental ill health. More insomnia was positively associated physical ill health. Reduced sleep quality and greater loneliness were associated with mental ill health. More sleep problems were positively associated with mental ill health. |
| Schiltz et al., 2020 | USA | Quantitative descriptive (self-report questionnaire) | 69 (56/13/NR) | 17-29, 20.24 (2.77) | Quantitative study of relationships among loneliness, mental health, autism features and social contact, using self-report questionnaire | The more autism features autistic adults reported, the more social and family loneliness, higher levels of anxiety and social depression. |
| Smith & Sharp, 2013 | UK | Qualitative (instant messaging semi-structured interviews) | HFA (high-functioning autism)/AS (Asperger syndrome) = 9 (NR/NR/NR) | 25-49, NR, NR | Semi-structured interviews on sensory experiences. | Unique sensory experiences cause  sensory stress, and they are  positively associated with isolation. |
| Southby & Robinson, 2018 | UK | Qualitative (semi-structured interviews) | HFASD = 14 (12/2/NR), family members = 3, volunteer mentors = 2, professionals = 11 | NR, NR, NR | Semi-structured interviews on the efficacy of “low-level” support. | Providing advocacy information and mentoring had significantly positive impacts on adults “with HFASD”. |
| Sundberg, 2018 | Hungary | Quantitative descriptive (self-report online questionnaire) | ASD = 85 (49/36/NR), control = (32/34/NR) | 14-69, 26.68, 10.78 | Self-report questionnaire on online gaming, loneliness and friendships. | Adult “with ASD” who play online games have more friends than those who do not, however, friendship quality or having a best/close friend is not associated with online gaming. Low to moderate use of online games is associated with less loneliness. |
| Syu & Lin, 2018 | Taiwan | Quantitative descriptive (self-report questionnaire) | 70 (46/24/NR) | 20-39, 27.8, 5.0 | Self-report questionnaire on sensory profile, anxiety and loneliness. | Sensory sensitivity was associated with higher level of anxiety and loneliness. |
| Van der Aa et al., 2016 | Netherlands | Mixed methods (online survey) | ASC = 113 (62/49/NR), control = 72 (28/44/NR) | ASC = NR, 40.2, 12.3, control = NR, 40.5, 12.1 | Online survey of computer-mediated communication (CMC) among people “with ASC”. | People “with ASC” use CMC more frequently, and they report high-level of satisfaction in online social life and CMC use is negatively associated with life satisfaction. |
| Van Hees et al., 2015 | Belgium | Qualitative (semi-structured interviews) | 23 (17/6/NR) | 18-25, NR, NR | Interviews on the experiences in higher education. | Students “with ASD” in higher education reported many challenges including social relationships, and they also reported the benefits because of their condition as well as their need for support. |

Note: NR = not reported, NA = not applicable, terminology to describe autism corresponds to the exact terms in each article and it is not the intention of the review.

| Appendix E. | | | | | | | | | | | | |
| --- | --- | --- | --- | --- | --- | --- | --- | --- | --- | --- | --- | --- |
| *Characteristics of the autistic participants* | | | | | | | | | | | | |
| **Study** | **Participants’ autism diagnoses** | | **Way of communication (verbal/non-verbal)^[[2]](#footnote-2)^** | | **IQ** | | **Co-occurring diagnoses** | | **Living situation** | **Employment** | **Highest level of education** | **Cultural background/ethnicity** |
| Ashbaugh et al., 2017 | Autism spectrum disorder (ASD) | | Verbal | | Average or above average | | NR | | All living at parent’s home | NA | All are currently in university | NR |
| Baldwin & Costley, 2016 | Autistic disorder (81%), Asperger’s disorder (17%), PDD-NOS (2%) | | NR | | NR | | NR | | With parent(s) (47%), with partner (12%), alone (29%), other (12%) | NR | NR | Australian (68%), British/European (18%), other (14%) |
| Bourdeau, 2020 (Doctoral thesis) | | NR (described as ASD) | | NR | | NR | | NR | NR | NR | NR | NR (demographic variables were not collected due to institutional review board limitations) |
| Brooks, 2014 (Doctoral thesis) | High functioning autism spectrum disorder (HFASD) | | NR | | Female: WASI-II VCI score (SD) = 107.0 (14.8), WASI-II PRI score (SD) = 98 (18.8)  Male: WASI-II VCI score (SD) = 105.7 (20.7), WASI-II PRI score (SD) = 102.5 (14.3) | | Female: mood disorder (n=19), anxiety disorder (n=8), ADHD (n=11), other psychiatric diagnoses (n=3)  Male: mood disorder (n=13), anxiety disorder (n=5), ADHD (n=10), other psychiatric diagnoses (n=0) | | Female: independently/with partner (n=14), with parents/family (n= 14)  Male: independently/with partner (n=7), with parents/family (n= 21) | NR | NR | NR |
| Caruana et al., 2021 | NR (described as autism) | | NR | | NR | | Study 1: NR  Study 2:  Depression (n=14), anxiety (n=12), post-traumatic stress disorder (n=6), obsessive-compulsive disorder (n=4), bipolar disorder (n=4), dyslexia (n=2), ADHD (n=2), Dyspraxia (n=1), other (n=6) | | NR | NR | NR | NR |
| Chen et al., 2016 | “High-functioning” autism (HFA) (n=5), Asperger’s syndrome (n=25) | | NR | | Minimum reading comprehension ability. (Australia sample: ≥ 85 on the reading comprehension subset of Woodcock Reading Mastery Test-3^rd^ edition (WRMT-III, Woodock, 2011), Taiwan sample: verbal IQ ≥ 70 on the Wechsler Adult Intelligent Scale-IV (Wechsler, 2008).) | | ADHD (n=3), depression (n=1), anxiety (n=1), OCD (n=2), depression and anxiety (n=3), depression, anxiety and OCD (n=1) | | Alone (n=3), with partner/children (n=3), with parents/siblings (n=21), with others (n=3) | Student (n=11), unemployed (n=8), part-time (n=3), full-time (n=7), volunteer work (n=1) | High school (n=8), certificate (n=5), diploma (n=2), Associate’s degree (n=1), Bachelor’s degree (n=10), Master’s degree (n=4) | Australian (n=14), Taiwan (n=16) |
| Ee et al. 2019 | Autism or autism spectrum Disorder/condition (n=49), autistic disorder (n=1), Asperger’s disorder (n=132), PDD-NOS (n=1), infantile autism (n=1), high functioning autism (n=33), missing (n=3) | | NR | | Basic proficiency in English | | NR | | Living alone (n=60, 27.5%), living with partner (n=100, 45.9%), living with parents (n=31, 14.2%), living with relatives (n=3, 1.4%), living with others (n=20, 9.2%), other arrangement (n=4, 1.8%), missing (n=2) | Employed (n=118, 44.3%) (They also reported ‘missing’ on which further details were not provided.) | Below year 12 (n=11, 5.1%), Year 12 (n=12, 5.6), further education (n=19, 89.2%), missing (n=6) (Many of their participants appeared not to have reported on this. The study was not clear what ‘missing’ meant and how it was different from participants’ not reporting the information.) | NR |
| Elmose 2020 | NR (described as autism) | | NR | | NR | | NR | | Living with no external support (n=8), living with some degree of external support (n=3), living at home (n=1), not disclosed (n=13) | Retired (n=1), early retirement (n=1), full-time (n=1), part-time (n=1), Work-testing, internship (n=2), sick-leave (n=1), unemployed (n=3), not disclosed (n=15) | Elementary schooling, grades 9–10 (n=1), secondary education (n=2), other youth education (n=3), higher education, short (n=2), higher education, long (n=4), currently in higher educational setting (n=3), not disclosed (n=10) | NR |
| Gantman et al., 2012 | Autistic disorder (n=4), Asperger’s Disorder (n=11), pervasive developmental disorder not otherwise specified (PDD-NOS) (n=2) | | NR | | Composite IQ score of greater than 70 on the Kaufman Brief Intelligence Test—Second Edition (KBIT-2; Kaufman and Kaufman 2005) | | NR | | With caregivers (including parents) (n=16) | NA | All are currently attending university at least part-time | Caucasian (n=10), Asian (n=5), Hispanic/Latino (n=2) |
| Han et al. 2019 | NR (described as ASD) | | Verbal IQ ≥80, verbal fluency based on Autism Diagnostic Observation Schedule, 2^nd^ edition | | Reading level ≥5th grade | | NR except that autistic participants did not have depression, psychotic or bipolar disorders and substance use disorders. | | NR | NR | NR | NR |
| Hedley et al., 2018a | ASD (n=11, 15.5%), Asperger’s syndrome (n=54, 76.1%), HFA (n=5, 7.0%), not specified (n=1, 1.4%) | | NR | | NR | | NR | | With spouse or partner (n=7, 9.9%), independent (n=15, 21.1%), with family member, relative or carer (n=47, 66.2%), other (n=2, 2.8%) | Part-time or full-time (n=37, 52.1%), no employment (n=34, 47.9%) | Primary school (n=1, 1.4%), some secondary (n=3, 4.2%), completed secondary (n=24, 33.8%), certificate (n=13, 18.3%), diploma (n=8, 11.3%), Bachelor’s degree (n=18, 25.4%), other (n=4, 5.6%) | Australian (n=60, 84.5%), Aboriginal or Torres Strait Islander (n=1, 1.4%), other (n=6, 8.5%), prefer not to say (n=4, 5.6%) |
| Hedley et al., 2018b | ASD (n=46, 24.9%), Asperger’s syndrome (n=110, 59.5%), HFA (n=23, 12.4%), autistic disorder (n=4, 2.2%), PDD-NOS (n=2, 1.1%) | | NR | | NR | | NR | | With parents (n=59, 31.9%), with relatives (n=4, 2.2%), with others (n=12, 6.5%), alone (n=38, 20.5%), as a couple (n=63, 34.1%), other (n=9, 4.9%) | Part-time or full-time (n=92, 49.7%) | Current secondary (n=14, 7.6%), some secondary (n=7, 3.8%), completed secondary (n=17, 9.2%), certificate or diploma (n=39, 21.1%), Bachelor’s degree (n=51, 27.6%), postgraduate degree (n=35, 18.9%), other/not reported (n=22, 11.9%) | NR |
| Hickey et al., 2018 | Asperger’s syndrome (n=8), HFA (n=5) | | Verbal in English | | NR | | No diagnosis of intellectual disabilities | | With spouse, children or partner (n=3), with family members (n=2), independent (n=4), supported housing (n=3), private house share (n=1) | Part-time or full-time (n=5), retired, unemployed (n=8) | Some secondary school (n=5), some university (n=1), Bachelor’s degree (n=6), Master’s degree (n=1) | NR |
| Hillier et al., 2018 | NR (described as autism spectrum disorder) | | NR | | NR | | NR | | NR | NA | All are currently in university | NR |
| Hull et al., 2017 | Autism/autistic disorder, Asperger Syndrome/Asperger’s Disorder, Autism Spectrum Disorder, Atypical Autism or PDD-NOS | | NR | | NR | | NR | | NR | NR | NR | British (n=41), North American (n=16), Western European (=15), other (n=10) |
| Hwang et al., 2017 | Asperger’s syndrome (n=13), ASD (n=1), “High functioning” autism (n=1) | | NR | | NR | | Mild intellectual disability (n=2), moderate intellectual disability (n=1) | | NR | NR | NR | NR |
| Jackson et al., 2018 | Asperger’s (n=34, 60.7%), ASD (n=10, 17.9%), HFA (n=8, 14.3%), PDD-NOS (n=4, 7.1%) | | NR | | NR | | NR | | NR | NR | NR | White (n=45, 80.4%), Hispanic (n=2, 3.6%), Black (n=1, 1.8%), Asian (n=4, 7.1%), mixed/other (n=4, 7.1%) |
| Jantz, 2011 | Asperger syndrome | | NR | | NR | | NR | | Alone (n=17, 48.5%), with parents (n=10, 28.6%), with roommates or spouse (n=6, 17.1%), with a dependant (n=1, 2.9%), community home (n=1, 2.9) | Full-time (n=13, 37.1%), part-time or self-employed (n=6, 11.4%), receiving social security disability insurance (n=3, 5.7%), retired (n=2, 2.9%), unemployed (n=9, 25.7%) | Postgraduate degree (n=12, 34.3%), some postgraduate school (n=3, 8.6%), Bachelor’s degree (n=7, 20.0%), Associate’s degree (n=2, 5.7%), some university (n=8, 22.9%), high school diploma or GED (n=3, 8.6%) | NR |
| Levinson, 2020 | NR (described as ASD) | | NR | | NR | | NR | | NR | NR | NR | White (n=101, 82%), Latinx (n=6, 5%), Asian (n=2, 2%), Black/African (n=2, 2%), Middle East/North Africa (n=1, 1%), Native American (n=1, 1%), multiracial (n=7, 6%), not reported (n=3, 2%) |
| Lin & Huang, 2017 | NR (described as ASD) | | NR | | NR | | Psychiatric disorders (n=15, 22.7%) | | NR | Full-time or part-time (n=30, 45.5%) | High school and below (n=16, 24.2%), university and above (n=30, 45.5%) | NR |
| Mazurek, 2013 | Autism or autistic disorder (29.6%), Asperger’s Disorder (63.9%), PDD-NOS (6.5%) | | NR | | NR | | NR | | NR | Full-time or part-time (49.1%), unemployed (26.9%) | NR | Caucasian (88.0%) |
| Mazurek, 2014 | Autism or autistic disorder (29.6%), Asperger’s Disorder (63.9%), PDD-NOS (6.5%) | | NR | | NR | | NR | | Alone (19.4%), with parents (38.0%), with partner, spouse or roommate (28.7%), other (13.9%) | Full-time (24.1%), part-time (25.0%), student (24.1%), unemployed (26.9%) | NR | Caucasian (88.0%) |
| McVey et al., 2016 | NR (described as ASD) | | NR | | M(SD) of Verbal IQ on the Kaufman Brief Intelligence Test – Second Edition (KBIT-2; Kaufman and Kaufman 2004): 93.38 (22.95) | | NR | | NR | NR | NR | Experimental group:  Caucasian (n=20)  Waitlist group:  Caucasian (n=21) |
| Merkler, 2007 | NR (described as young adults with “high-functioning” autism) | | NR | | 69-118 (M=93.13, Sd=12.59) on BETA III (Kellogg & Morton, 1999) | | NR | | NR | NR | NR | Caucasian (n=33, 89%), African American (n=1, 3%), Asian (n=1, 3%), Indian (n=2, 5%) |
| Milton & Sims, 2016 | NR (described as adults on the autism spectrum) | | NR | | NR | | NR | | NR | NR | NR | NR |
| Orsmond et al., 2013 | NR (described as ASD) | | NR | | NR | | NR | | With a parent/guardian (82.0%), alone/with a roommate (7.8%), under supervision (10.2%) | Full-time or part-time (33.5%) | Currently attending postsecondary school (12.4%) | White (70.0%), African American (18.7%), Mixed/other (11.3%) |
| Russell et al., 2020 | NR (described as autism spectrum disorder) | | NR | | NR | | NR | | NR | NR | NR | NR |
| Schiltz et al., 2020 | NR (described as Autism) | | NR (they reported the participants were predominantly verbal) | | M(SD)=96.56(17.79), ranging from 63-145 on Kaufman Brief Intelligence Test, Second Edition (KBIT-2) (Kaufman & Kaufman, 2004) | | NR | | NR | NR | NR | White (85.50%), Asian (5.80%), Black (4.40%), Middle Eastern (1.40%), American Indian (1.40%), not reported (1.40%) |
| Smith & Sharp, 2013 | Asperger Syndrome | | NR | | NR | | NR | | Alone (n=4), with a spouse, partner and/or children (n=3), with parents (n=2) | Full-time or part-time (n=3), in training for employment (n=3), unemployed (n=3) | GCSEs (n=2), A-levels (n=4), Bachelor’s degree (n=2), degree plus professional qualification (n=1) | White British (n=8), Ashkenzai Jewish (n=1) |
| Southby & Robinson, 2018 | ASD or autism (56.3%), others are not speficied | | NR | | NR | | NR | | NR | NR | NR | NR |
| Sundberg, 2018 | HFASD | | NR | | NR | | NR | | NR | NR | NR | NR |
| Syu & Lin, 2018 | Pervasive developmental disorders, Asperger’s disorder, or PDD-NOS | | NR | | IQ ≥ 70 (measure used to determine this was not reported), reading comprehension and writing skills in Mandarin Chinese | | NR | | NR | Employed (n=33, 47.1%) | High school and below (n=16, 22.9%), college and above (n=54, 77.1%) | NR |
| Van der Aa et al., 2016 | NR (described as “high-functioning” autism spectrum disorders) | | NR | | NR | | NR | | Independent (n=93, 84.7%), non-independent (with parents, sheltered etc.) (n=17, 15.3%) | Paid employment (n=42, 37.8%), retired (n=2, 1.8%), student (n=13, 11.7%), disability allowance (n=32, 28.8%), unemployed and actively seeking (n=6, 5.4%), not employed otherwise (n=16, 14.4%) | Primary school (n=1, 9%), lower vocational/intermediate secondary education (n=11, 9.9%), intermediate vocational/higher secondary education (n=21, 18.9%), higher vocational education (n=35, 31.5%), university (n=43, 38.7%) | NR |
| Van Hees et al., 2015 | Autistic disorder, Asperger’s syndrome, or PDD-NOS | | NR | | NR | | NR | | NR | NA | All are currently attending university | NR |
| Note: NA = not applicable, NR = not reported, the Table reports the description exclusively on autistic participants though some studies include both autistic and non-autistic participants. | | | | | | | | | | | | |

| Appendix F. | | | | | | | |
| --- | --- | --- | --- | --- | --- | --- | --- |
| *Characteristics of Loneliness measures identified in the included studies* | | | | | | | |
| **Measures** | **Aim of tool** | **Target population** | **Number of items**  **(Subscales)** | **Response options** | **Format** | **Used in what references?** | **Validity and reliability** |
| The UCLA Loneliness Scale, Version3 (Russell, 1996) | To measure loneliness and social isolation | General population | 20  Note: Hillier et al. (2018) states they used the scale that is 10 questions, however, the other included studies and the original study suggest 20 items. | 4-point Linkert scale | Self-report questionnaire | Brooks (2014)*, Hedley et al. (2018a), Hillier et al. (2018), Jantz (2011), Russel (2020)* | Validated and reliable in neuro-typical university students with Cronbach’s alpha of .89-.94 (Russell, 1996). Test-retest reliability (r=.73) was reported in three studies (Brooks, 2014, Hillier et al., 2018, Jantz, 2011). |
| Social and Emotional Loneliness Scale for Adults (SELSA) (DiTommaso & Spiner, 1993) | To measure social, family and romantic loneliness | Adults in the general population | 37  (social, family and romantic loneliness) | 7-point scale Linkert scale | Self-report questionnaire | Bourdeau (2020), Gantman et al. (2012), McVey et al. (2016), Merkler (2007), Schiltz et al. (2020) | Validated and reliable with the Cronbach’s alpha of .89 to .93 (DiTommaso & Spiner, 1993). Internal consistency within the study (0.71) was reported in one study (McVey et al., 2016). |
| The UCLA Loneliness Scale Short Form (ULS-8) (Hays & DiMatteo, 1987) | To measure loneliness and social isolation | General population | 8 | 4-point Linkert scale | Self-report questionnaire | Ee et al. (2019)*, Hedley et al. (2018b), Lin & Huang (2017)*, Mazurek (2013), Mazurek (2014), Sundberg (2017)*, Syu & Lin (2018) | Validated and reliable with Cronbach’s alpha of .84 (Hays & DiMatteo, 1987). |
| Revised UCLA Loneliness Scale (Russell et al., 1980) | To measure loneliness | General population | 20 | 4-point Linkert scale | Self-report questionnaire | Caruana et al. (2021), Levinson (2020)* | Cronbach’s alpha ranged between .917-.930 among participants with ASD, Cronbach’s alpha ranged between .930 - .944 among neurotypical participants (Levinson, 2020) |
| 3 item UCLA Loneliness Scale (Hughes et al., 2004) | To measure loneliness in a telephone survey with a large numbers of sample | General population | 3 | 3-point Linker scale | Self-report questionnaire | Jackson et al. (2018) | Validated and reliable with the good internal consistency despite the Cronbach’s alpha being .72 (Hughes et al., 2004) |
| Isolation and Affect measure (Merkler, 2007) based on the Peer Network and Peer Dyadic Loneliness Scale (PNPDL) (Hoza, Bukowski, & Berry, 2000) | To measure loneliness | Adults “with high-functioning autism” and the neuro-typical university students | 28  (social network isolation, dyadic isolation) | 5-point Linkert scale | Self-report questionnaire | Merkler (2007)* | Reported as validated and reliable with Cronbach’s alpha ranged from .97 and .98 in autistic adults (Merkler, 2007) |
| Loneliness Scale based on the Revised UCLA loneliness scale (Russell et al., 1980) | To scale loneliness as one of the aspects of life that affects life satisfaction in adults “with high-functioning ASC” | Adults “with ASC” | 6  *based on the 20 items used in the original scale (Russell, Peplau, &  Cutrona, 1980) | 5-point scale Linkert scale | Self-report questionnaire | Van der Aa et al. (2016) | NR, but the Revised UCLA Loneliness Scale was validated and reliable with Cronbach’s alpha of .94 (Russell et al., 1980). |
| Loneliness in context questionnaire (LiCQ) (Asher & Weeks, 2014) | To measure loneliness for adults in daily contexts | Adults in the general population | 10 | 5-point Linkert scale | Self-report questionnaire | Han et al. (2019)* | Cronbach’s alpha of .90, .88, .87 for TD control, ASD, and TD currently depressed groups were reported in the study, validated and reliable (Asher & Weeks, 2014) |
| Note: NR = not reported, neuro-typical = non-autistic, Cronbach’s alpha is the measure of internal consistency and shows the reliability, close to 1.0 being more reliable.  Note: Asterisks indicate the studied that reported the scores in both autistic and non-autistic comparison groups. | | | | | | | |

References

Allely, C. (2015). Experiences of prison inmates with autism spectrum disorders and the knowledge and understanding of the spectrum amongst prison staff: a review. *Journal of Intellectual Disabilities and Offending Behaviour, 6*(2), 55-67. <https://doi.org/10.1108/jidob-06-2015-0014>

Bailey, K. M., Frost, K. M., Casagrande, K., & Ingersoll, B. (2020). The relationship between social experience and subjective well-being in autistic college students: A mixed methods study [Article]. *Autism: The International Journal of Research & Practice, 24*(5), 1081-1092. <https://doi.org/10.1177/1362361319892457>

Bal, V. H., Wilkinson, E., White, L. C., Law, J. K., Consortium, S., Feliciano, P., & Chung, W. K. (2021). Early Pandemic Experiences of Autistic Adults: Predictors of Psychological Distress. *Autism research : official journal of the International Society for Autism Research*. <https://doi.org/https://dx.doi.org/10.1002/aur.2480>

Brooks, S., & Paterson, G. (2011, Jun 2011

2021-02-11). Using Contact Work in Interactions with Adults with Learning Disabilities and Autistic Spectrum Disorders. *British Journal of Learning Disabilities, 39*(2), 161-166. <https://doi.org/http://dx.doi.org/10.1111/j.1468-3156.2010.00643.x>

Buonaguro, E. F., & Bertelli, M. O. (2021, 2021

2021-03-01). COVID-19 and intellectual disability/autism spectrum disorder with high and very high support needs: issues of physical and mental vulnerability. *Advances in Mental Health and Intellectual Disabilities, 15*(1), 8-19. <https://doi.org/http://dx.doi.org/10.1108/AMHID-07-2020-0016>

Cage, E., Di Monaco, J., & Newell, V. (2018). Experiences of autism acceptance and mental health in autistic adults. *Journal of autism and developmental disorders, 48*(2), 473-484. <https://doi.org/https://doi.org/10.1007/s10803-017-3342-7>

Cai, R. Y., & Richdale, A. L. (2016, Jan 2016

2018-10-07). Educational Experiences and Needs of Higher Education Students with Autism Spectrum Disorder. *Journal of Autism and Developmental Disorders, 46*(1), 31-41. <https://doi.org/http://dx.doi.org/10.1007/s10803-015-2535-1>

Cassidy, S., Bradley, P., Robinson, J., Allison, C., McHugh, M., & Baron-Cohen, S. (2014). Suicidal ideation and suicide plans or attempts in adults with Asperger's syndrome attending a specialist diagnostic clinic: a clinical cohort study. *The Lancet Psychiatry, 1*(2), 142-147. <https://doi.org/https://doi.org/10.1016/S2215-0366(14)70248-2>

Causton-Theoharis, J., Ashby, C., & Cosier, M. (2009). Islands of loneliness: Exploring social interaction through the autobiographies of individuals with autism. *Intellectual and Developmental Disabilities, 47*(2), 84-96. <https://doi.org/https://doi.org/10.1352/1934-9556-47.2.84>

Churchard, A., Ryder, M., Greenhill, A., & Mandy, W. (2019). The prevalence of autistic traits in a homeless population [Article]. *Autism: The International Journal of Research & Practice, 23*(3), 665-676. <https://doi.org/10.1177/1362361318768484>

Connor, A., Sung, C., Strain, A., Zeng, S., & Fabrizi, S. (2020, Jun 2020

2020-06-27). Building Skills, Confidence, and Wellness: Psychosocial Effects of Soft Skills Training for Young Adults with Autism. *Journal of Autism and Developmental Disorders, 50*(6), 2064-2076. <https://doi.org/http://dx.doi.org/10.1007/s10803-019-03962-w>

Feldhaus, C., Koglin, U., Devermann, J., Logemann, H., & Lorenz, A. (2015, 2015

2021-02-11). Students with Autism Spectrum Disorders and Their Neuro-Typical Peers--Differences and Influences of Loneliness, Stress and Self-Efficacy on Life Satisfaction. *Universal Journal of Educational Research, 3*(6), 375-381. <https://search.proquest.com/scholarly-journals/students-with-autism-spectrum-disorders-their/docview/1720058483/se-2?accountid=14511>

<https://ucl-new-primo.hosted.exlibrisgroup.com/openurl/UCL/UCL_VU2?url_ver=Z39.88-2004&rft_val_fmt=info:ofi/fmt:kev:mtx:journal&genre=article&sid=ProQ:ProQ%3Aeric&atitle=Students+with+Autism+Spectrum+Disorders+and+Their+Neuro-Typical+Peers--Differences+and+Influences+of+Loneliness%2C+Stress+and+Self-Efficacy+on+Life+Satisfaction&title=Universal+Journal+of+Educational+Research&issn=23323205&date=2015-01-01&volume=3&issue=6&spage=375&au=Feldhaus%2C+Carmen%3BKoglin%2C+Ute%3BDevermann%2C+Jens%3BLogemann%2C+Hanna%3BLorenz%2C+Alfred&isbn=&jtitle=Universal+Journal+of+Educational+Research&btitle=&rft_id=info:eric/EJ1066253&rft_id=info:doi/>

Friedman, L., Sterling, A., Leann Smith, D., & Mailick, M. R. (2019, Oct 2019

2019-09-19). Conversational Language Is a Predictor of Vocational Independence and Friendships in Adults with ASD. *Journal of Autism and Developmental Disorders, 49*(10), 4294-4305. <https://doi.org/http://dx.doi.org/10.1007/s10803-019-04147-1>

Furuhashi, Y., & Furuhashi, S. (2015). The effect of group cognitive behavior therapy on adults with high-functioning autism spectrum disorder. In *Autism Spectrum Disorders: Early Signs, Intervention Options and Family Impact* (pp. 103-127). <https://www.scopus.com/inward/record.uri?eid=2-s2.0-84958832626&partnerID=40&md5=d78ed900db4c826b36f835488dfd83cb>

Happe, F., & Charlton, R. A. (2012). Aging in autism spectrum disorders: a mini-review. *Gerontology, 58*(1), 70-78. <https://doi.org/https://dx.doi.org/10.1159/000329720>

Hong, Q. N., Pluye, P., Fàbregues, S., Bartlett, G., Boardman, F., Cargo, M., Dagenais, P., Gagnon, M.-P., Griffiths, F., & Nicolau, B. (2018). Mixed methods appraisal tool (MMAT), version 2018. *Registration of copyright, 1148552*, 10.

Howlin, P., Goode, S., Hutton, J., & Rutter, M. (2004). Adult outcome for children with autism. *Journal of child psychology and psychiatry, and allied disciplines, 45*(2), 212-229. <http://ovidsp.ovid.com/ovidweb.cgi?T=JS&PAGE=reference&D=med5&NEWS=N&AN=14982237>

Kapp, S. K., Gantman, A., & Laugeson, E. A. (2011). *Transition to Adulthood for High-Functioning Individuals with Autism Spectrum Disorders*. <Go to ISI>://WOS:000386854200026

Kaufman, A. S., & Kaufman, N. L. (2004). *Kaufman Brief Intelligence Test, second edition (KBIT-2)* Pearson.

Kellogg, C., & Morton, N. (1999). *Beta III*. Psychological Corporation San Antonio.

Kirby, A. V. (2016, May 2016

2018-10-06). Parent Expectations Mediate Outcomes for Young Adults with Autism Spectrum Disorder. *Journal of Autism and Developmental Disorders, 46*(5), 1643-1655. <https://doi.org/http://dx.doi.org/10.1007/s10803-015-2691-3>

Koegel, L. K., Ashbaugh, K., Koegel, R. L., & Detar, W. J. (2013, November 2013

2016-09-27). Increasing Socialization In Adults With Asperger's Syndrome. *Psychology in the Schools, 50*(9), 899-909. <https://doi.org/http://dx.doi.org/10.1002/pits.21715>

Laugeson, E. A., Gantman, A., Kapp, S. K., Orenski, K., & Ellingsen, R. (2015, Dec 2015

2018-10-05). A Randomized Controlled Trial to Improve Social Skills in Young Adults with Autism Spectrum Disorder: The UCLA PEERS<sup></sup> Program. *Journal of Autism and Developmental Disorders, 45*(12), 3978-3989. <https://doi.org/http://dx.doi.org/10.1007/s10803-015-2504-8>

Lewis, L. F. (2016). Exploring the Experience of Self-Diagnosis of Autism Spectrum Disorder in Adults. *Archives of Psychiatric Nursing, 30*(5), 575-580. <https://doi.org/https://dx.doi.org/10.1016/j.apnu.2016.03.009>

McConkey, R., Cassin, M. T., McNaughton, R., & Armstrong, E. (2021). Enhancing the social networks of adults with ASD: a low level community intervention [Article]. *Advances in Autism*. <https://doi.org/10.1108/AIA-07-2020-0043>

McVey, A. J. (2016). *A replication and extension of the peers<sup>®</sup> for young adults social skills intervention* (Publication Number 10037450) [M.S., Marquette University]. ProQuest Dissertations & Theses Global. Ann Arbor. <https://search.proquest.com/dissertations-theses/replication-extension-peers-sup->®-young-adults/docview/1775227185/se-2?accountid=14511

<https://ucl-new-primo.hosted.exlibrisgroup.com/openurl/UCL/UCL_VU2?url_ver=Z39.88-2004&rft_val_fmt=info:ofi/fmt:kev:mtx:dissertation&genre=dissertations+%26+theses&sid=ProQ:ProQuest+Dissertations+%26+Theses+Global&atitle=&title=A+replication+and+extension+of+the+peers%C2%AE+for+young+adults+social+skills+intervention&issn=&date=2016-01-01&volume=&issue=&spage=&au=McVey%2C+Alana+J.&isbn=978-1-339-54804-3&jtitle=&btitle=&rft_id=info:eric/&rft_id=info:doi/>

McVey, A. J., Dolan, B. K., Willar, K. S., Pleiss, S., Karst, J. S., Casnar, C. L., Caiozzo, C., Vogt, E. M., Gordon, N. S., & Van Hecke, A. V. (2016, 2016/12/01). A Replication and Extension of the PEERS® for Young Adults Social Skills Intervention: Examining Effects on Social Skills and Social Anxiety in Young Adults with Autism Spectrum Disorder. *Journal of autism and developmental disorders, 46*(12), 3739-3754. <https://doi.org/https://doi.org/10.1007/s10803-016-2911-5>

Morrison, K. E., DeBrabander, K. M., Jones, D. R., Ackerman, R. A., & Sasson, N. J. (2020, Nov). Social Cognition, Social Skill, and Social Motivation Minimally Predict Social Interaction Outcomes for Autistic and Non-Autistic Adults. *Frontiers in Psychology, 11*, Article 591100. <https://doi.org/10.3389/fpsyg.2020.591100>

Simmons, A. L. (2020, 2020/07/02). COVID-19 social distancing: A snippet view of the autistic social world. *Disability & Society, 35*(6), 1007-1011. <https://doi.org/https://doi.org/10.1080/09687599.2020.1774866>

Spain, D., & Blainey, S. H. (2015, Oct 2015

2021-02-11). Group Social Skills Interventions for Adults with High-Functioning Autism Spectrum Disorders: A Systematic Review. *Autism: The International Journal of Research and Practice, 19*(7), 874-886. <https://doi.org/http://dx.doi.org/10.1177/1362361315587659>

Stice, L. V. B., & Lavner, J. A. (2019). Social Connectedness and Loneliness Mediate the Association Between Autistic Traits and Internalizing Symptoms Among Young Adults [Article]. *Journal of Autism and Developmental Disorders, 49*(3), 1096-1110. <https://doi.org/10.1007/s10803-018-3812-6>

Wendler, D. (2019). Improv theater as a social cognition intervention for autism [Health & Mental Health Treatment & Prevention 3300]. *Dissertation Abstracts International: Section B: The Sciences and Engineering, 80*(8-B(E)), No-Specified. <http://ovidsp.ovid.com/ovidweb.cgi?T=JS&PAGE=reference&D=psyc16&NEWS=N&AN=2019-41141-135> (Dissertation Abstracts International)

1. The criteria numbers in the table (e.g., S1., 1.1.) correspond to those of the MMAT tool Hong, Q. N., Pluye, P., Fàbregues, S., Bartlett, G., Boardman, F., Cargo, M., Dagenais, P., Gagnon, M.-P., Griffiths, F., & Nicolau, B. (2018). Mixed methods appraisal tool (MMAT), version 2018. *Registration of copyright, 1148552*, 10. . [↑](#footnote-ref-1)
2. Almost all the entries are coded NR because the papers did not report participants’ method of communication. Where information on communication cannot be inferred from what was written in the articles, the entries were coded NR. Whilst it could be assumed that the participants were verbal because the data were collected via interviews, interviews can be possible without verbal communication (e.g., text-based chat). [↑](#footnote-ref-2)
